# Geometrically Confined Strain Engineering of MoS_2_ via Quasi‐Van Der Waals Recrystallization of Gold Nanopillars

**DOI:** 10.1002/advs.202506488

**Published:** 2025-08-11

**Authors:** Kyungmin Yang, Yuna Lee, Dongjoon Rhee, Bongjun Choi, Adam Alfieri, Marija Drndić, Deep Jariwala, Gwan‐Hyoung Lee

**Affiliations:** ^1^ Department of Materials Science and Engineering Seoul National University Seoul 18826 South Korea; ^2^ Department of Electrical and Systems Engineering University of Pennsylvania Philadelphia PA 19104 USA; ^3^ Department of Physics and Astronomy University of Pennsylvania Philadelphia PA 19104 USA

**Keywords:** 2D materials, funnel effect, gold nanopillars, purcell effect, strain engineering

## Abstract

Transition metal dichalcogenides (TMDs) are promising materials for next‐generation electronics due to their atomically thin body and exceptional optoelectronic properties. Their ultrathin and stiff nature make them highly sensitive to strain, enabling modulation of lattice and band structures and enhancing carrier mobility via tensile strain. While uniform strain degrades optical properties of MoS_2_ due to the indirect bandgap, localized strain enhances them through the funnel effect, highlighting the importance of localized strain engineering. In this study, localized strain is achieved in monolayer MoS_2_ using geometrically confined quasi‐van der Waals (qvdW) recrystallization of patterned gold nanopillars. After transferring hBN‐encapsulated MoS_2_ onto the pillars and annealing, the gold recrystallizes into thicker, more crystalline structures, inducing ≈0.15% local tensile strain. This results in a 65‐fold increase in photoluminescence (PL) intensity due to the funnel effect, Fabry‐Pérot interference, and Purcell effect. Additionally, field‐effect mobility is significantly enhanced to 100 cm^2^V^−1^s^−1^, a two‐order of magnitude improvement. The work shows a way to apply local strain in MoS_2_ using geometrically confined gold pillars via qvdW recrystallization, offering a possibility for advanced optoelectronic devices.

## Graphical abstract

Geometrically confined quasi‐van der Waals (qvdW) recrystallization of patterned gold induces localized tensile strain in monolayer MoS_2_, enhancing its optoelectronic performance. The strain, induced during annealing after hBN encapsulation, leads to a 65‐fold increase in photoluminescence and a two‐order magnitude boost in mobility, offering a strategy for strain‐engineered 2D optoelectronic devices.

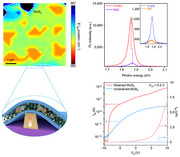

## 1 Introduction

As silicon‐based devices are approaching their physical limits and the challenges associated with further miniaturization request alternatives, transition metal dichalcogenides (TMDs) have gained significant attention as potential candidates in advanced electronic and optoelectronic devices owing to their atomic‐scale thinness and exceptional electrical and optical properties.^[^ ^1^, ^2^, ^3^ ^]^ Except for these superior properties, high sensitivity of TMDs strain offers more efficient ways to modify their lattice and band structures, such as the funneling effect,^[^ ^4^, ^5^ ^]^ piezoelectric effect,^[^ ^6^ ^]^ and reduced phonon scattering.^[^ ^7^, ^8^ ^]^ It was recently reported that tensile strain applied to MoS_2_ in the range of 0.2–2% strongly enhance the carrier mobility by a factor ranging from 1.6 to 100.^[^ ^9^, ^10^, ^11^, ^12^, ^13^, ^14^, ^15^, ^16^, ^17^, ^18^, ^19^ ^]^ Therefore, several methods to generate strain in TMDs have been proposed: deposition of stressors,^[^ ^19^ ^]^ and stretching or bending of the supporting substrates.^[^ ^12^, ^18^ ^]^ However, these methods to generate uniform strain in TMDs induce narrowing of K−Γ indirect gap, leading to photoluminescence (PL) quenching,^[^ ^12^, ^20^ ^]^ while spatially confined strain enhances the PL intensity and improves the electrical properties.^[^ ^14^ ^]^ Previous studies indicate that a tensile strain exceeding 0.66% in monolayer MoS_2_ induces a direct‐to‐indirect bandgap transition, whereas a localized strain below this threshold enhances PL intensity via the funneling effect.^[^ ^14^, ^20^, ^21^, ^22^, ^23^ ^]^ Consequently, the development of techniques not only to precisely control the magnitude of tensile strain but also to spatially manipulate its localization is crucial for effectively enhancing the optical and electrical properties of TMD materials.

In this study, we show strain engineering of MoS_2_ through spatially confined qvdW recrystallization of gold nanopillars. The hBN‐encapsulated MoS_2_ was transferred onto a patterned gold nanopillars. During annealing, the metal patterns agglomerate into single crystal pillars with increased height, inducing local tensile strain of ≈ 0.15% in the MoS_2_ as we reported previously on qvdW recrystallization of gold.^[^ ^24^ ^]^ This spatially confined hBN‐encapsulated MoS_2_ with high strain showed significantly improved PL intensity by a factor of 65 owing to the funneling effect, Fabry‐Pérot interference, and Purcell enhancement. Additionally, we achieved a high field‐effect mobility of 100 cm^2^V^−1^s^−1^ in monolayer MoS_2_ field‐effect transistors owing to suppressed electron‐phonon scattering,^[^ ^25^ ^]^ a two‐order of magnitude improvement over unstrained MoS_2_. Our strain engineering and unique structure of hBN‐encapsulated MoS_2_ on gold pillars are effective in modification of electrical and optical properties of TMDs.

## 2 Results and Discussion

**Figure 1:**
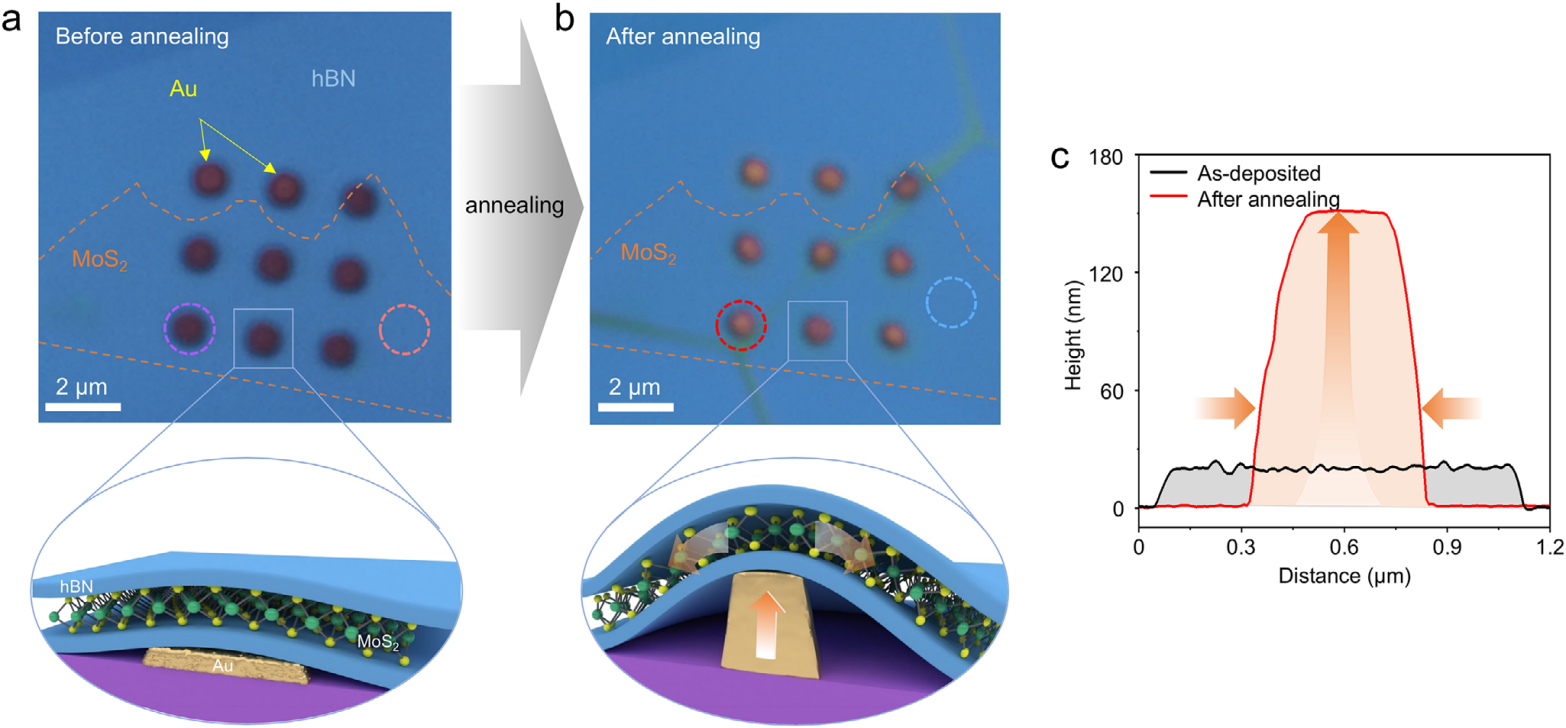
Optical microscopic images and schematics of qvdW recrystallization process (a) before annealing and (b) after annealing to induce tensile strain on MoS_2_. The dashed circles in (a) and (b) indicate the regions where the Raman spectra of MoS_2_ were obtained (Figure 2a): hBN‐encapsulated MoS_2_ on SiO_2_ before annealing (MS, orange circle), hBN‐encapsulated MoS_2_ on gold nanopillar before annealing (MG, purple circle), hBN‐encapsulated MoS_2_ on SiO_2_ after annealing (A‐MS, blue circle), and hBN‐encapsulated MoS_2_ on recrystallized gold pillars after annealing (A‐MG, red circle). c) AFM height profiles of an as‐deposited gold nanopillar (black) and a recrystallized gold pillar (red).

**Figure** **1**a,b shows optical images and schematics of qvdW recrystallization process. First, an array of gold nanopillars with a thickness of 20 nm and diameter of 1 µm was patterned on a SiO_2_/Si substrate by e‐beam evaporation and lithography. Then, monolayer MoS_2_ encapsulated with hBN was transferred onto these gold nanopillars (Figure 1a). After annealing, the gold nanopillars were agglomerated and recrystallized into single crystal pillars (Figure 1b), as we previously reported.^[^ ^24^ ^]^ The height of gold pillars was significantly increased to ≈150 nm along with a smooth top surface of gold as shown in the height profiles measured by atomic force microscopy (AFM) in Figure 1c and Figure S1 (Supporting Information), which induces spatially controlled tensile strain in the hBN‐encapsulated MoS_2_.

In this study, we use specific abbreviations to describe the different experimental configurations for clarity: hBN‐encapsulated MoS_2_ on a SiO_2_ before annealing (MS), hBN‐encapsulated MoS_2_ on gold nanopillars before annealing (MG), hBN‐encapsulated MoS_2_ on SiO_2_ after annealing (A‐MS) and hBN‐encapsulated MoS_2_ on recrystallized gold pillars after annealing (A‐MG).

**Figure 2:**
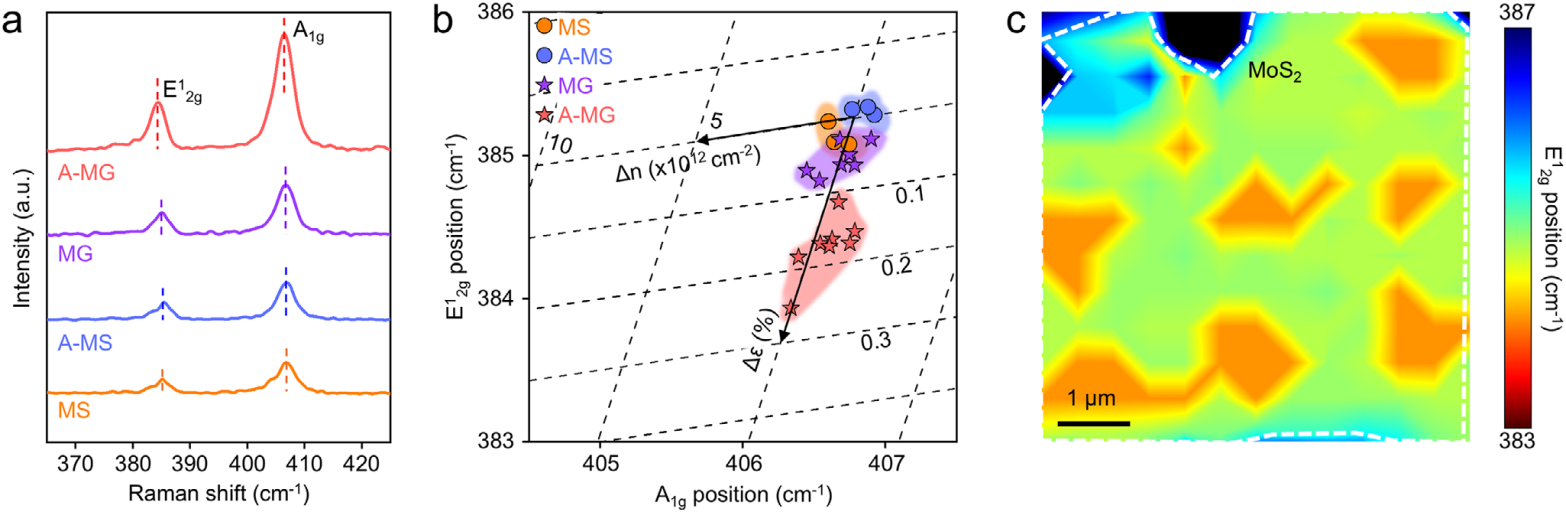
a) Raman spectra of hBN‐encapsulated MoS_2_ on SiO_2_ before annealing (MS, orange), hBN‐encapsulated MoS_2_ on gold nanopillar before annealing (MG, purple), hBN‐encapsulated MoS_2_ on SiO_2_ after annealing (A‐MS, blue), and hBN‐encapsulated MoS_2_ on recrystallized gold pillars after annealing (A‐MG, red). The color of these Raman spectra match the color of the dashed circles in Figure 1a,b, indicating the regions from which they were obtained. b) Plot showing the correlation between the E^1^_2g_ − A_1g_ peak positions for all four samples. Shifts in the E^1^_2g_ peak position indicate the generation of tensile strain in MoS_2_. c) Spatial map of the E^1^_2g_ peak position that reveals the strain distribution in annealed MoS_2_ on the gold nanopillars.

To investigate the effect of recrystallization‐induced geometric changes of patterned gold on local strain in MoS_2_, we analyzed the Raman spectra of hBN‐encapsulated MoS_2_ on SiO_2_ and gold nanopilllar before and after annealing (**Figure** **2**a). In A‐MG, the redshift of the E^1^_2g_ peak compared to MS is observed, while the A_1g_ peak shows no noticeable shift. Since the E^1^_2g_ peak is sensitive to strain and A_1g_ peak is sensitive to doping, this spectral change indicates that the recrystallized gold particles induce the tensile strain in MoS_2_ with minimal changes in doping levels. To quantify the strain induced by the recrystallized gold, we plotted the positions of the E^1^_2g_ and A_1g_ peaks obtained from MoS_2_ on each gold array in Figure 2b.^[^ ^26^, ^27^ ^]^ The Raman spectra of MS represented two prominent peaks with E^1^_2g_ at 385.1 cm^−1^ and A_1g_ at 406.7 cm^−1^.^[^ ^28^ ^]^ The MG exhibited the E^1^_2g_ peak at 384.9 cm^−1^ and the A_1g_ peak at 406.7 cm^−1^ with no noticeable shift in the A_1g_ peak compared to MS, which indicates that the as‐deposited gold film does not affect the effect of doping on MoS_2_ due to the hBN encapsulation. The slight redshift of ≈ 0.2 cm^−1^ in the E^1^_2g_ peak suggested that the as‐deposited gold film induced a weak tensile strain of 0.023 ± 0.025% compared to MS when the hBN‐encapsulated MoS_2_ was transferred to the 20 nm thick gold nanopillar.^[^ ^29^, ^30^, ^31^ ^]^ The E^1^_2g_ peak of A‐MG showed a further redshift to 384.3 cm^−1^, representing a 0.8 cm^−1^ shift compared to MS. After annealing, the points shifted along the strain (∆ε) axis, indicating that tensile strain increased to 0.150 ± 0.036% for A‐MG. Previous studies have reported that ≈1% strain causes a 5.2 cm^−1^ redshift in the E^1^_2g_ peak, which aligns well with our results.^[^ ^32^, ^33^ ^]^ This result revealed that the tensile strain is amplified on the recrystallized gold pillar than on the as‐deposited gold nanopillar because this method induced strain in situ from geometrical changes, simultaneously with metal recrystallization. As the thickness of the gold increases during annealing, the hBN‐encapsulated MoS_2_ can conformally rise. To further clarify the effect of our in situ strain engineering approach, we also investigated the Raman peaks of hBN/MoS_2_ transferred onto the hBN/Au pillar, formed by recrystallizing the hBN‐encapsulated gold nanopillar prior to the transfer process (Figure S2, Supporting Information). The hBN/MoS_2_ transferred on the recrystallized gold pillar (transferred A‐MG) exhibited an average tensile strain of 0.055%, significantly lower than the 0.15% strain achieved via the in situ strain induction method. The lower strain for transferred A‐MG is a result of the hBN/MoS_2_ conforming less effectively to the underlying topography compared to the A‐MG case. Figure 2c represents a mapping image of the E^1^_2g_ peak position of MoS_2_, visualizing the spatial distribution of strain. The regions showing redshifts of the E^1^_2g_ mode spatially coincide with the gold nanopillars as shown in Figure 2a, demonstrating that the qvdW recrystallization can induce significant strain in 2D materials on individual gold nanopillars in a controlled manner.

**Figure 3:**
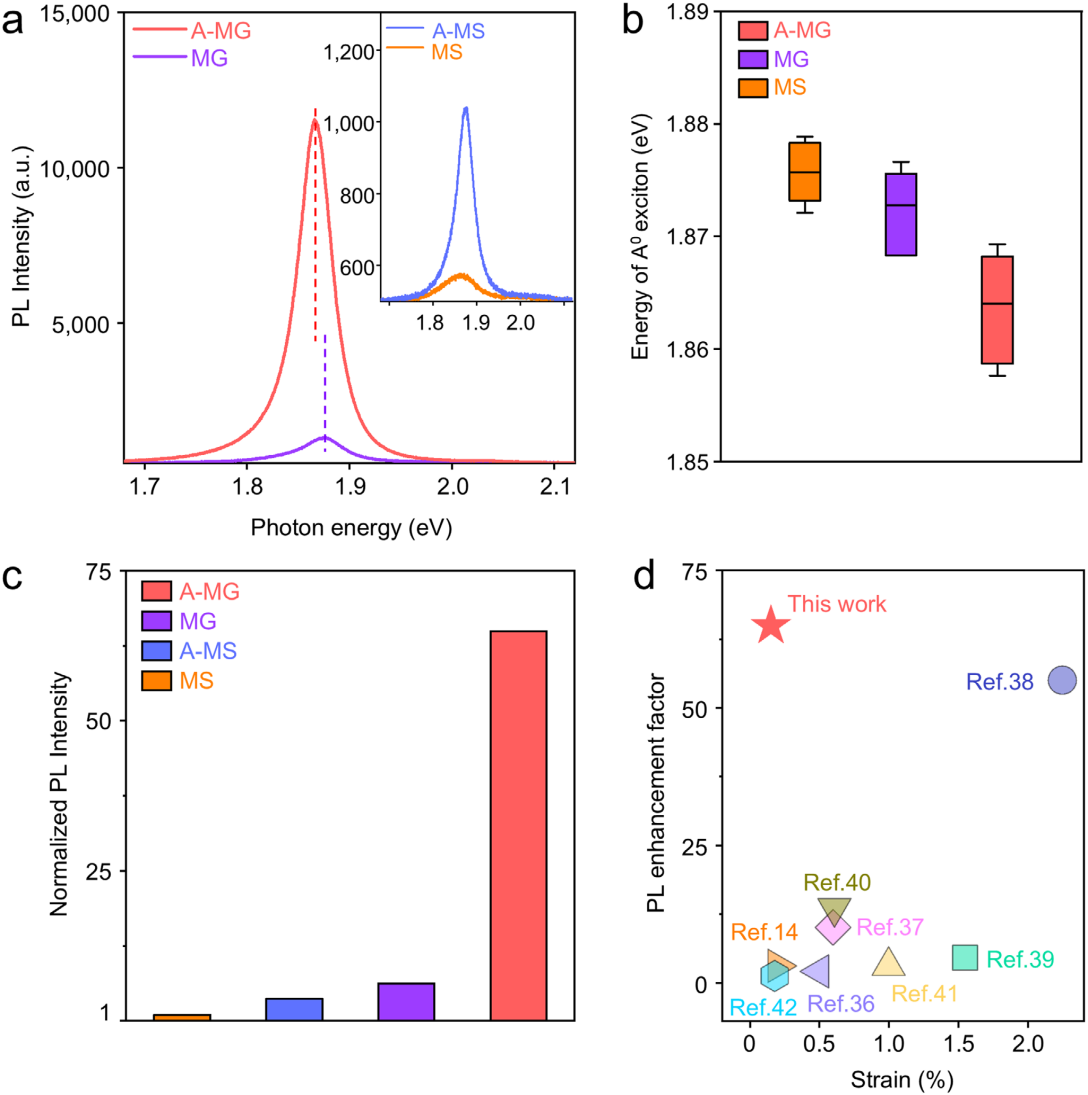
a) PL spectra of hBN‐encapsulated MoS_2_ on recrystallized gold pillars after annealing (A‐MG, red) and hBN‐encapsulated MoS_2_ on gold nanopillar before annealing (MG, purple). The inset shows PL spectra of hBN‐encapsulated MoS_2_ on SiO_2_ before annealing (MS, orange) and hBN‐encapsulated MoS_2_ on SiO_2_ after annealing (A‐MS, blue). b) PL peak positions of the A^0^ exciton in A‐MG, MG, and MS. For statistical analysis, PL spectra were collected from five different samples. The mean is represented by the center lines in boxes, the boxes contain 25–75th percentiles of the dataset and whiskers represented minimum and maximum values. c) PL intensities of four samples in (a). For comparison, PL intensities were normalized to that of MS. d) Comparison of the PL enhancement factors for A‐MG (this work) and monolayer MoS_2_ under various strains reported in previous studies.

To study the optical properties of strain‐induced MoS_2_, we measured the PL spectra of MoS_2_ (**Figure** **3**a). In A‐MG, a significant increase in PL intensity and a redshift in the PL peak were observed. This phenomenon was noted even before recrystallization (MG), suggesting that the recrystallized single crystal gold particles not only induce strain but also enhance the optical properties of MoS_2_. To further investigate the origin of these PL spectral changes, we plotted the peak position of the A^0^ excitonic emission, which is the main component of the MoS_2_ PL spectra, as depicted in Figure 3b. The detailed deconvolution of the spectra for each sample is provided in Figure S3 (Supporting Information). Before annealing, the A^0^ exciton peak of MG was redshifted by 2.9 meV compared to MS. This redshift is attributed to a slight tensile strain of ≈ 0.023% induced by transferring hBN‐encapsulated MoS_2_ on the as‐deposited gold nanopillar. In contrast, the A^0^ exciton peak for A‐MG showed a higher redshift of 12.0 meV relative to MS due to the 0.15% tensile strain induced by the recrystallized gold pillar. Since the PL peak originates from the excitonic transition occurring at the direct bandgap of MoS_2_, the redshift of the A^0^ exciton peak indicates a reduction in bandgap due to tensile strain.^[^ ^32^, ^34^ ^]^ Therefore, we deduced that the bandgap becomes narrower in the region of MoS_2_ strained by the gold nanopillar compared to the surrounding flat regions, which drives exciton funneling toward gold nanopillar area. This funneling effect can lead to exciton concentration in the strain area, thus enhancing PL. For further comparison, we normalized the PL intensity obtained from each position of MoS_2_ to the PL intensity of MS, as shown in Figure 3c. The PL intensity of A‐MG exhibited 65‐fold enhancement than that of MS. While the funneling effect plays a major role in this enhancement, the hBN encapsulation annealing carried out during the recrystallization process also contributes to tailoring the optical properties of MoS_2_. As shown in the inset of Figure 3a and summarized in Figure 3c, the PL intensity of A‐MS increased by 3.7 times compared to MS along with a blue shift in the PL peak due to the de‐doping effect, which agrees with previous reports.^[^ ^26^, ^35^ ^]^ The de‐doping effect is induced by hBN encapsulation annealing, which leads to the substitution of chalcogen atoms in the encapsulated TMD materials with atoms such as oxygen. It has been reported that de‐doping of MoS_2_ suppresses the formation of trions, which act as non‐radiative recombination pathways, thereby enhancing the PL intensity by ≈3.5 times and causing a blue shift in the PL peak.^[^ ^26^ ^]^ Figure 3d shows a benchmark graph^[^ ^14^, ^36^, ^37^, ^38^, ^39^, ^40^, ^41^, ^42^ ^]^ comparing the PL enhancement factor (*I*_strained_ / *I*_unstrained_, where *I* is the PL intensity) achieved in this study (A‐MG) with previous reports as a function of strain. A notable feature of our work is that a significant PL enhancement is observed even under relatively small strain compared to previous studies. It suggests that, aside from exciton funneling induced by local strain in MoS_2_, additional factors contribute to this enhancement. The substantial increase in PL intensity of A‐MG might arise from several factors: the funneling effect caused by spatially controlled tensile strain, hBN‐encapsulation annealing, and the plasmonic effect of the recrystallized Au. We confirmed the main factors responsible for the PL enhancement in our work by performing additional characterization. When hBN/MoS_2_ was transferred on pre‐formed gold pillars covered with hBN (Transferred A‐MG), small tensile strain of ≈0.055% was applied to MoS_2_, leading to small redshift of 2.5 meV in A^0^ exciton peak (Figure S4a, Supporting Information). Notably, the PL intensity of transferred MoS_2_ on the pre‐formed gold pillars increased by a factor of 7.05 compared to the same MoS_2_ sample placed on SiO_2_ (MS), although this PL enhancement was not as high as that of A‐MG (Figure 3a). This enhanced PL is attributed to the plasmonic effect of the recrystallized gold. The gold surface beneath the bottom hBN spacer enables a Purcell enhancement effect, facilitating the spontaneous emission enhancement of the A^0^ exciton through plasmonic resonance coupling.^[^ ^43^ ^]^

**Figure 4:**
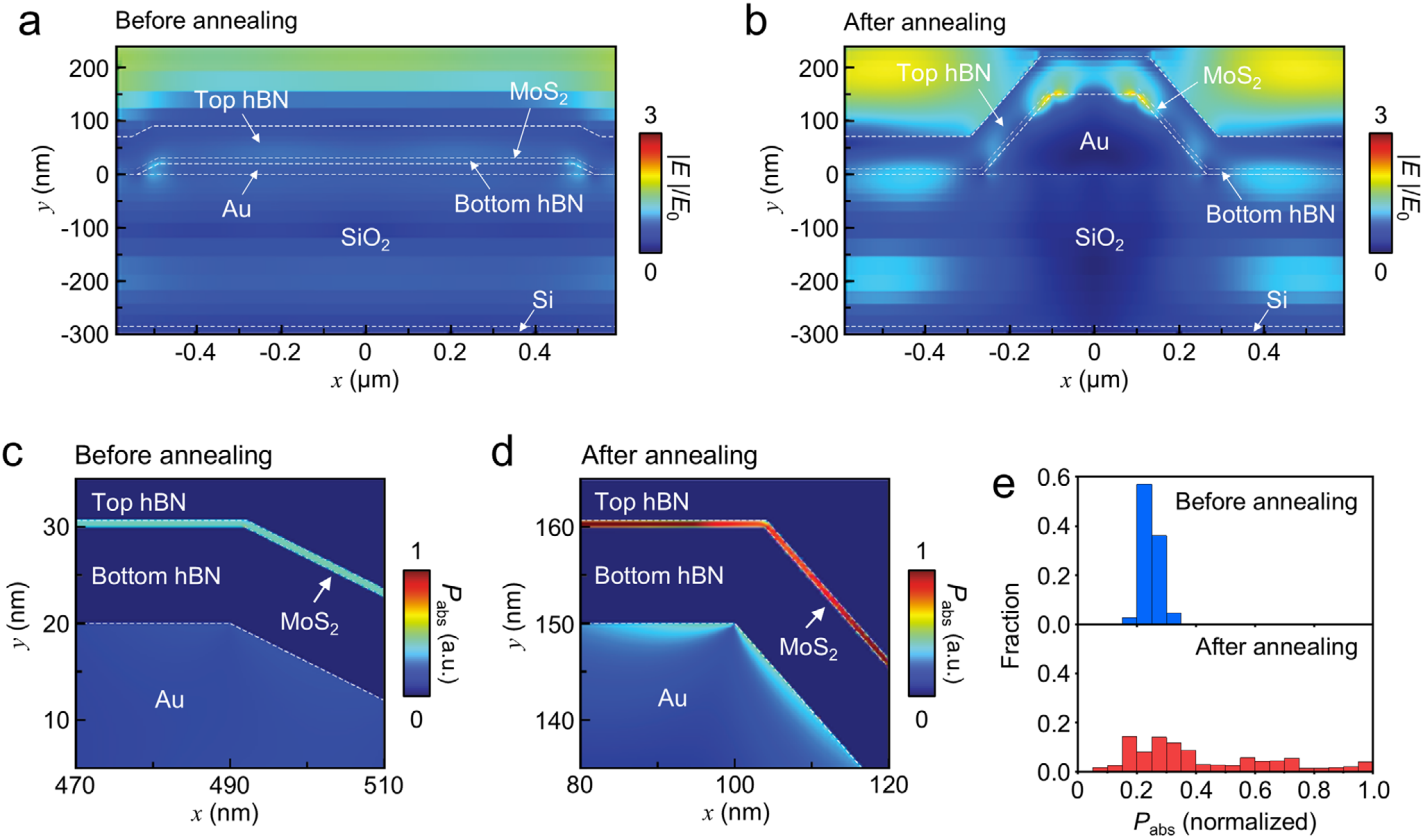
Spatial distribution of electric fields calculated using finite‐difference time‐domain (FDTD) simulations for a) MG and b) A‐MG. Spatial distribution of the light power absorption per unit volume (*P*_abs_) for c) MG and d) A‐MG. The *P*_abs_ values are normalized to the maximum observed in A‐MG. e) Histogram showing the statistical distribution of *P*_abs_ for MG (blue) and A‐MG (red).

The strong and localized electric fields on recrystallized gold pillar can increase the exciton generation and recombination rate in MoS_2_. Finite‐difference time‐domain (FDTD) simulations confirm the distribution of electric fields in transferred MoS_2_ on as‐deposited and recrystallized gold surfaces as shown in **Figure** **4**a,b. After recrystallization, the electric field is strongly enhanced at the localized edge regions of the gold pillar compared to before annealing as shown in Figure 4a,b and Figure S11 (Supporting Information). The enhanced electric field is prominently attributed to the higher localized surface plasmon resonance, which results from changes in geometry and crystallinity of the gold. According to Fermi's golden rule, the intensified electric field strengthens the light‐matter interaction, thereby increasing the optical transition probability, which in turn boosts the light absorption in MoS_2_.^[^ ^44^ ^]^ As a result, the light power absorption per unit volume (P_abs_) of MoS_2_, calculated from the electric field intensity (|E|^2^), showed a notable increase after annealing as shown in Figure 4c,d and Figure S8 (Supporting Information). The overall light absorption in MoS_2_ (*P*_abs_ integrated over the MoS_2_ region) increased by a factor of 1.72 after annealing (Figure 4e), demonstrating that the electric field enhancement associated with the gold nanopillar increases optical absorbance and exciton generation. Moreover, under the plasmonic conditions, an emitter in an excited state placed near a metal nanoparticle can more efficiently relax to a lower energy level via radiative processes. This enhancement arises from the sharp increase in the final photonic density of states at the resonance wavelength, which offers a strong and accessible decay channel.^[^ ^44^, ^45^ ^]^ The greater availability of final states at this energy enhances the optical transition probability, ultimately leading to an increased spontaneous emission rate. Furthermore, the Fabry‐Pérot interference at bottom hBN/Au interface can induce multiple reflections of incident light, thereby boosting the optical absorbance of MoS_2._^[^ ^46^ ^]^ Transfer matrix method (TMM) simulation based on Maxwell's equations confirmed this effect, showing amplified absorbance at wavelengths near the energies of A and B excitons (Figure S12, Supporting Information).

Finally, to determine which factor—strain‐induced exciton funneling, the Purcell effect from gold pillars, or Fabry‐Pérot interference—plays the most significant role in PL intensity enhancement, we compared the PL intensity of A‐MG and A‐MS. As shown in Figure S4b (Supporting Information), the A‐MG exhibits a 17.76‐fold increase in PL intensity compared to A‐MS. Since both samples were measured after annealing, the PL enhancement can be analyzed without considering de‐doping effects. The A‐MG experiences an additional 0.2% tensile strain compared to A‐MS as shown in Figure 2b. According to previous reports, the same level of strain applied to monolayer MoS_2_ resulted in a threefold increase in PL intensity.^[^ ^14^ ^]^ Given that A‐MG exhibits a significantly higher PL intensity (≈ 18‐fold) than A‐MS, it can be inferred that the Purcell effect induced by gold pillars and Fabry‐Pérot interference play a more dominant role in PL enhancement than exciton funneling. In conclusion, the structure employed in this work not only enhances PL intensity through strain engineering but also benefits from multiple synergistic effects. This suggests that our approach provides a promising platform for optoelectronic applications based on 2D materials.

**Figure 5:**
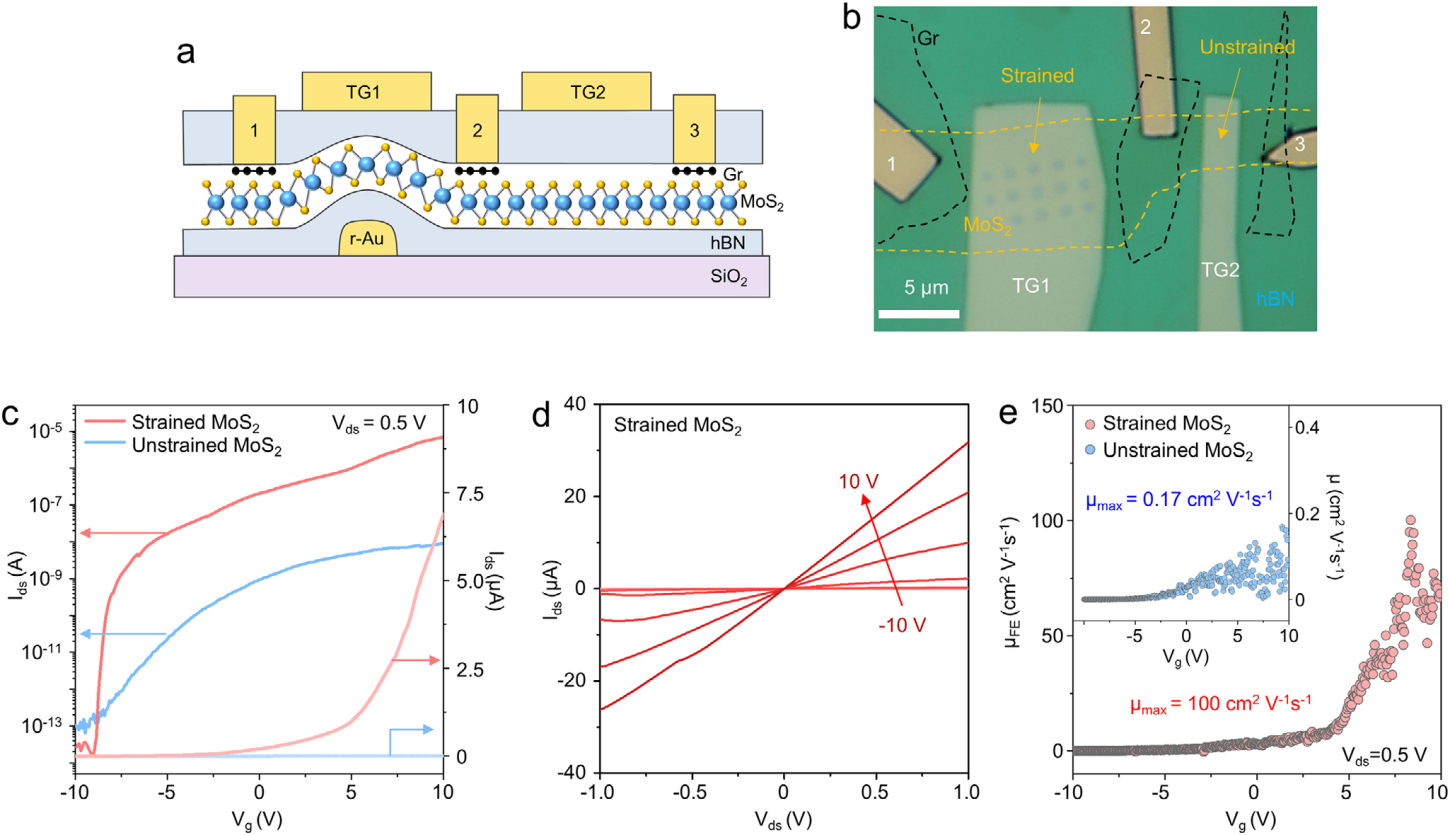
Schematic illustration (a) and optical microscopy image (b) of fluorinated graphene contact devices with strained and unstrained MoS_2_. c) Transfer curves (I_ds_‐V_g_) obtained from strained (red) and unstrained MoS_2_ (blue). d) Output curves (I_ds_‐V_ds_) of strained MoS_2_. e) FET mobility (µ) of strained (red) and unstrained MoS_2_ (blue) at room temperature (300 K).

To investigate electrical properties of strained MoS_2_, we fabricated two field‐effect transistors (FETs) from hBN‐encapsulated MoS_2_, one on SiO_2_ and the other on a recrystallized gold pillar, as shown in the schematic in **Figure** **5**a and the optical microscopic image of Figure 5b. Monolayer graphene (electrodes), monolayer MoS_2_, and hBN were subsequently transferred onto the gold nanopillars using pick‐up technique, followed by recrystallization process via annealing. The source and drain electrodes were fabricated on graphene regions using the fluorinated graphene via contact method established in our previous work.^[^ ^47^ ^]^ Figure 5c shows the transfer curve (I_ds_‐V_g_) of the A‐MG (strained) and A‐MS (unstrained) regions of MoS_2_. The unstrained MoS_2_ device was characterized by an on‐current (I_on_) of ≈10^−9^ A with an on/off ratio of 10^4^. In contrast, the strained MoS_2_ exhibited an I_on_ of 10^−5^ A and an on/off ratio of 10^8^. The linear output characteristic of the strained MoS_2_ is shown in Figure 5d. Figure 5e presents filed‐effect mobilities (µ_FE_ = (dI_ds_/dV_g_) L/(WC_gate_V_ds_), where L and W is channel length and width respectively and C_gate_ ≈ 5.54862 × 10^−4^ F m^−2^ is the capacitance per unit area of the ≈60 nm thick hBN used as the gate dielectric) of the two devices, plotted as a function of gate voltage (V_g_). The peak mobility of the strained MoS_2_ was 100 cm^2^V^−1^s^−1^, two‐orders of magnitude higher than that of the unstrained counterpart (0.17 cm^2^V^−1^s^−1^, the inset of Figure 5e), which might be due to strain‐induced quenching of electron‐phonon scattering.^[^ ^25^ ^]^ Previous studies have shown that the greater the applied strain, the larger the K‐Q valley separation, reducing electron‐phonon intervalley scattering and increasing mobility.^[^ ^48^ ^]^ Moreover, it has been reported that tensile strain in MoS_2_ induces lattice distortion, leading to lattice polarization. This lattice symmetry breaking increases the dielectric constant of MoS_2_, thereby enhancing the carrier mobility.^[^ ^49^ ^]^

**Figure 6:**
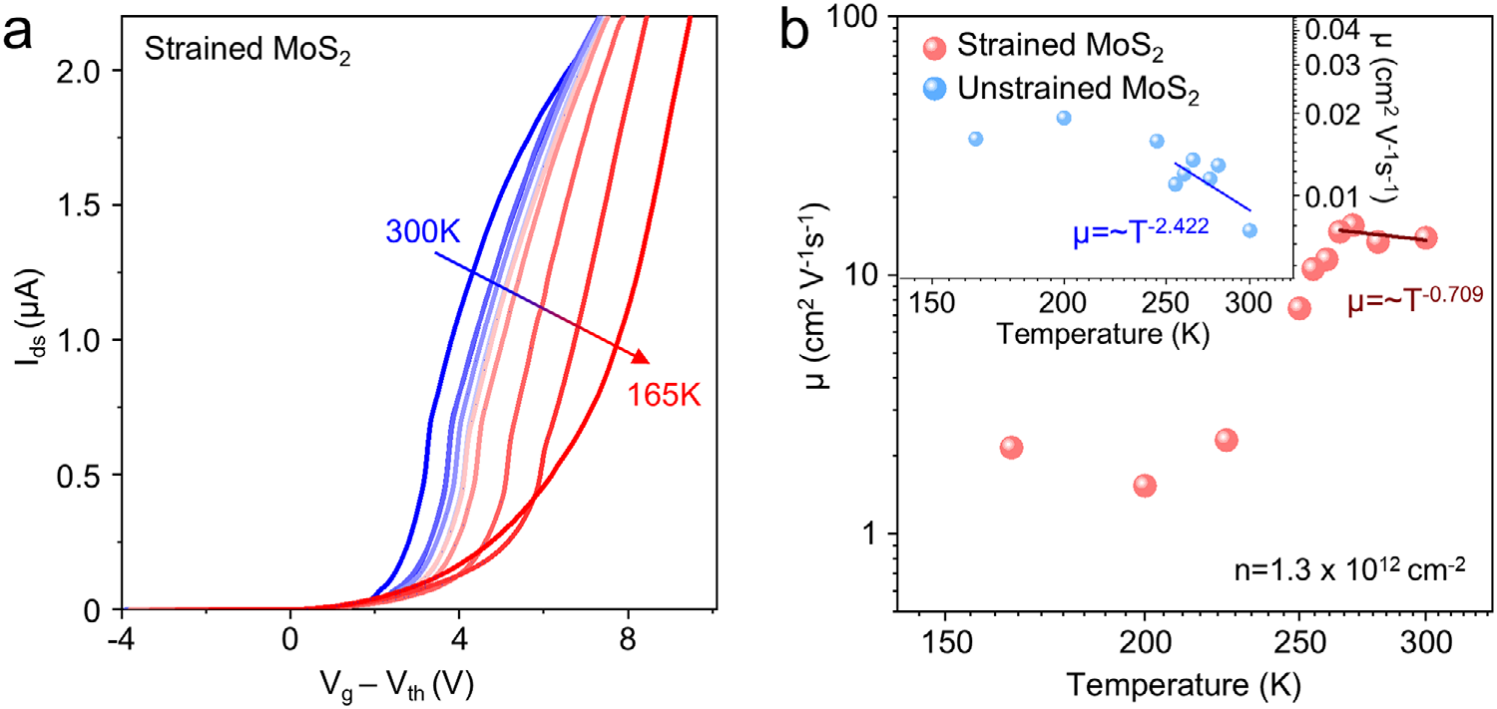
a) Temperature‐dependent transfer curves of strained MoS_2_. b) FET mobility (µ) of strained (red) and unstrained MoS_2_ (blue) as a function of temperature at a carrier density of 1.3 × 10^12^ cm^−2^.

To confirm the primary scattering mechanisms in strained MoS_2_ devices, we fabricated devices with the same structure as in Figure 5a and measured the transfer curve of strained (**Figure** **6**a; Figure S14, Supporting Information) and unstrained (Figure S15, Supporting Information) MoS_2_ devices over a temperature range from 300 K (room temperature) to 165 K. To account for shifts in threshold voltage (*V_th_*), we compared the FET mobility at the same carrier density using the equation *n*  = *C_gate_* (*V_g_* − *V_th_*)/*q*, where *n*, *C_gate_*, and *q* are the carrier density, the gate dielectric capacitance, and the elementary charge, respectively and V_th_ was defined as the voltage at which I_DS_ reaches 10^−10^ A (Figure 6b). In 2D materials like TMDs, charge transport is typically dominated by phonon scattering at high temperatures and charge impurity scattering at low temperatures. However, the charge transport mechanisms in our strained MoS_2_ showed distinct differences. By fitting the mobility as a function of temperature ($\mu ∼T^{-\gamma }$) in Figure 6b, unstrained MoS_2_ exhibited *γ* ∼ 2.422, indicating that optical phonon scattering dominated charge transport in unstrained MoS_2_.^[^ ^50^, ^51^ ^]^ In contrast, the strained MoS_2_ showed *γ* ∼ 0.709 at high temperatures, indicating that charge impurity scattering is dominant. This is attributed to the quenching of phonon scattering by tensile strain. The temperature dependence of mobility further supports that our method is highly effective, demonstrating an increase in mobility by two orders of magnitude.

## 3 Conclusion

In conclusion, we have demonstrated an approach for achieving enhancment of electrical and optical properties of monolayer MoS_2_ using a quasi‐van der Waals epitaxial recrystallization of gold nanopillars. These recrystallized gold nanopillars enable the application of a tensile strain of 0.15% to the 1L‐MoS_2_. The recrystallized gold not only induces localized tensile strain, causing exciton funneling, but also enables resonant plasmonic excitation resulting in a 65‐fold increase in PL intensity in MoS_2_. In addition, our strained MoS_2_ FET exhibits a significantly enhanced mobility due to the suppression of electron‐phonon scattering, resulting in superior electrical performance. Our strained MoS_2_ FET exhibits a field effect mobility of 100 cm^2^ V^−1^ s^−1^ at a bias 0.5 V, which represents a two‐order of magnitude enhancement compared to the unstrained MoS_2_ FET on the same crystal flake. Therefore, our strain application technique not only enhances the optical properties of 2D materials but also improves their electrical characteristics, providing a novel design strategy for 2D material‐based electronics and optoelectronics.

## 4 Experimental Section

### Sample Preparation

To fabricate the gold crystal array, a polymethylmethacrylate (PMMA) resist film was patterned on the SiO_2_(285 nm)/Si using e‐beam lithography, deposited gold by e‐beam evaporator at the rate of 0.2 Å s^−1^ in a high vacuum condition (<10^−7^ Torr), and then performed lift‐off to remove the resist. Next, the mechanically exfoliated top‐hBN, MoS_2_, and bottom‐hBN with the dry pick‐up method was sequentially picked up^[^ ^52^ ^]^ using a polycarbonate (PC) film and then transfer them onto the patterned gold array. After the transfer process, the samples were dipped in chloroform for 1 h to remove the PC film, and annealed at 700 °C for 3 h under a vacuum of 10^−4^ Torr.

Material Characterization

The samples were examined by Raman spectroscopy (JASCO, NRS‐4500) with a 532 nm laser and AFM (Park Systems, NX10).

Device Fabrication and Electrical Measurement

We sequentially picked up the monolayer graphene flakes, which served as the electrode, MoS_2_ channel, and bottom hBN, using the top hBN. The top and bottom hBN layers used had approximate thicknesses of 60 and 10 nm, respectively. The hBN‐encapsulated MoS_2_ was transferred onto a pre‐patterned gold array, creating devices on two distinct regions: one region where the MoS_2_ is placed on the gold pattern and the other region where the MoS_2_ remains pattern‐free. To fabricate the fluorinated graphene contact, the top‐hBN/graphene region was patterned by e‐beam lithography, and treated the sample with XeF_2_ gas (SAMCO, VPE‐4F) to partially remove the top hBN and form fluorinated graphene. Then, Cr/Pd/Au was deposited with thicknesses of 1, 30, and 40 nm using an e‐beam evaporator. After lift‐off, the top gate electrode on top hBN was fabricated by depositing Cr/Au with thickneses of 10 and 20 nm, respectively. The electrical characteristics were measured under a vacuum condition using a probe station connected to a semiconductor parameter analyzer (Keithley, 4200A‐SCS). For the temperature dependent study, another device was fabricated following the same procedure, and measured the electrical characteristics while lowering the chamber temperature (Keithley 4200A).

Ellipsometry Measurements

The optical constants, particularly refractive index (*n*) and extinction coefficients (*k*), used to simulate SiO_2_ and as‐deposited gold were obtained from ellipsometry measurements, while those of Si,^[^ ^53^ ^]^ hBN,^[^ ^54^ ^]^ monolayer MoS_2_,^[^ ^55^ ^]^ and single‐crystalline gold^[^ ^56^ ^]^ were acquired from previous reports. Ellipsometry data (*Ψ* and *Δ*) were first collected from a SiO_2_/Si substrate at incident angles of 65°, 70°, and 75° over a light wavelength range of 400–1000 nm (Park systems, EP4). The measured *Ψ* and *Δ* were then fitted to a SiO_2_/Si bilayer model, in which the optical properties of the Si substrate were described by a built‐in model, while those of the SiO_2_ layer were represented by a Cauchy model. The fitted Cauchy parameters were used to determine *n* and *k* of the SiO_2_ layer. After establishing the model for the SiO_2_/Si substrate, a second set of ellipsometry measurements was performed on a SiO_2_/Si substrate with an as‐deposited gold film at incident angles of 55°, 65°, and 75° over the same wavelength range. To obtain *n* and *k* of the as‐deposited gold film, the resulting data were analyzed using an Au/SiO_2_/Si trilayer model, where only the fitting parameters for gold were adjusted, while those for SiO_2_/Si were kept fixed. Gold was modeled using a Drude oscillator to describe the optical response associated with free electrons and multiple Lorentz oscillators to account for interband transitions.

Finite‐Difference Time‐Domain Simulation

Spatial maps of electric fields and light power absorption were obtained from finite‐difference time‐domain (FDTD) simulations using commercial software (Ansys Lumerical FDTD, Ansys Inc.). To reveal the effect of the nanopillar and pillar structures on electric field enhancement and light absorption in MoS_2_ while minimizing computational costs, a 2D simulation was performed using a cross section of the hBN/MoS_2_/hBN/Au/SiO_2_/Si structure. The simulation box was divided into 0.05 nm × 0.05 nm mesh elements to effectively model the MoS_2_ layer. A total‐field scattered‐field (TFSF) source spanning 1.2 µm was used to inject a 532‐nm plane wave of light over the entire nanopillar‐structured region in the direction normal to the substrate. The polarization direction of light was parallel to the 2D simulation box plane. During simulation, the electric field at each point was recorded using a frequency‐domain profile monitor, which could be used to calculate the light power absorption per unit volume (*P*_abs_) according to the relation reported in a previous literature.^[^ ^57^ ^]^

Transfer Matrix Method (TMM) Simulation

The TMM is employed to calculate the absorbance in each layer of multilayer structures using a custom‐built Python code.^[^ ^58^, ^59^ ^]^ The structure hBN (10 nm) / monolayer MoS_2_ (0.7 nm) / hBN (t nm) / Au (100 nm) / Si is adopted, where the bottom hBN layer thickness (t) is varied from 0 to 100 nm to calculate the absorbance of the monolayer MoS_2_.

## Conflict of Interest

The authors declare no conflict of interest.

## Author Contributions

K.Y. and Y.L. contributed equally to this work. K.Y., Y.L., and G.H.L. designed and conceived this work. Y.L. fabricated the samples and performed the optical measurement. K.Y. fabricated devices and performed electrical measurements with help from M.D., D.R., A.A. and B.C. performed simulations with help from D.J. K.Y., Y.L. and G.H.L. wrote the paper together.

## Supporting information



Supporting Information

## Data Availability

The data that support the findings of this study are available from the corresponding author upon reasonable request.
