# Supplementary material for: Geometrically Confined Strain Engineering of MoS2 via Quasi‐Van Der Waals Recrystallization of Gold Nanopillars
Source: Adv Sci (Weinh). 2025 Aug 11;12(41):e06488. doi: 10.1002/advs.202506488 (PMC12591099; doi:10.1002/advs.202506488)
Supplement: Supplementary file 1 — Supporting Information [file ADVS-12-e06488-s001.docx]

**Supporting information**

**Geometrically confined strain engineering of MoS_2_ via quasi-van der Waals recrystallization of gold nanopillars**

*Kyungmin Yang^a,+^, Yuna Lee^a,+^, Dongjoon Rhee^b^, Bongjun Choi^b^, Adam Alfieri^b^, Marija Drndić^c^, Deep Jariwala^b^ and Gwan-Hyoung Lee^a^**

^a^ Department of Materials Science and Engineering, Seoul National University, Seoul 18826, Korea

^b^ Department of Electrical and Systems Engineering, University of Pennsylvania, Philadelphia, Pennsylvania 19104, United States

^c^ Department of Physics and Astronomy, University of Pennsylvania, Philadelphia, Pennsylvania 19104, United States

^+^ These authors contributed equally

* E-mail: gwanlee@snu.ac.kr

**Supplementary Notes**

**Note S1. Quantification of strain and doping in monolayer MoS_2_ using Raman spectroscopy**

In monolayer MoS_2_, the prominent in-plane E^1^_2g_ and out-of-plane A_1g_ Raman modes are sensitive to both strain and doping, as widely reported in the literature.^[1-7]^ These peaks exhibit shifts depending on the magnitude of strain and carrier concentration, and such shifts can be quantitatively described using the following equation:^[8-9]^

$$\left( \begin{matrix} \Delta Pos E_{2g}^{1} \\ \Delta Pos A_{1g} \end{matrix} \right)= \left( \begin{matrix} -2\gamma_{E_{2g}^{1}}Pos E_{2g}^{1} & k_{n,E_{2g}^{1}} \\ -2\gamma_{A_{1g}}Pos A_{1g} & k_{n,A_{1g}} \end{matrix} \right)\left( \begin{matrix} \varepsilon\\ n \end{matrix} \right)$$

Where $\gamma$ represents the Grüneisen parameter for each phonon mode, and $k_{n}$ is the charge doping coefficient. In this paper, we adopted previously reported Grüneisen parameter for biaxial strain and charge doping coefficient.^[10]^

$\gamma_{E_{2g}^{1}}=0.68$, $\gamma_{A_{1g}}=0.21$, $k_{n,E_{2g}^{1}}= \frac{0.33}{{10}^{13}}cm$, $k_{n,A_{1g}}= \frac{2.22}{{10}^{13}}cm$

This linear set of equations can be reformulated as follows.

$$\varepsilon=\frac{k_{n,A_{1g}}\Delta Pos E_{2g}^{1}-k_{n,E_{2g}^{1}}\Delta Pos A_{1g}}{2\gamma_{A_{1g}}PosA_{1g}k_{n,E_{2g}^{1}}-2\gamma_{E_{2g}^{1}}Pos E_{2g}^{1}k_{n,A_{1g}}}$$

$$n=\frac{\gamma_{A_{1g}}(\Delta Pos E_{2g}^{1})(Pos A_{1g})- \gamma_{E_{2g}^{1}}(\Delta Pos A_{1g})(Pos E_{2g}^{1})}{\gamma_{A_{1g}}Pos A_{1g} k_{n,E_{2g}^{1}}- \gamma_{E_{2g}^{1}}Pos E_{2g}^{1} k_{n,A_{1g}}}$$

By applying this expression, we can calculate the strain(ε) and charge carrier concentration(n) in a monolayer MoS_2_ by measuring the shift ($\Delta Pos E_{2g}^{1}, \Delta Pos A_{1g})$ in Raman peak positions with respect to those of a reference sample ($Pos E_{2g}^{1}, Pos A_{1g}$). It should be noted that the extracted strain and carrier concentration indicate the relative degree of strain and doping in the sample compared to the reference sample, and these values do not represent absolute quantities.

**Note S2. Advantages and disadvantages of differents methods for strain engineering**

| Method | Advantage | Disadvantage | References |
| --- | --- | --- | --- |
| Stressor film deposition | - Wafer-scale compatibility with CMOS process  - Enhanced electrical properties by strain engineering | - Limited uniformity in nanoscale patterning  - Reduction in PL intensity resulting from strain-mediated transition to indirect band structure in MoS_2_ | ^[10-12]^ |
| Substrate stretching/bending  (flexible substrates) | - Externally controllable dynamic strain  - Enhanced electrical properties by strain engineering | - Requirement for external mechanical setup  - Reduction in PL intensity resulting from strain-mediated transition to an indirect band structure in MoS_2_ | ^[13-15]^ |
| Transfer onto pre-patterned nanostructures | - Localized and precisely engineered strain applicability  - Improved electrical and optical properties induced by localized strain | - Complex alignment between MoS_2_ and underlying structures  - Interfacial slippage during post-transfer processing | ^[16-18]^ |
| Piezoelectric substrate actuation | - Electrically tunable and highly reproducible in-situ strain  - Enhanced electrical properties enabled by strain engineering | - Limited strain range  - Requirement for high voltage | ^[19-20]^ |
| Laser‑induced local heating | - Localized patterning with non-contact methodology  - Relatively simple implementation | - Risk of thermal damage to MoS_2_  - Strain instability | ^[21]^ |
| Our work | - Effective strain induction enabled by in-situ pillar recrystallization  - Improved electrical and optical properties induced by localized strain  - Significant optical enhancement achievable with minimal strain, enabled by localized strain and synergistic effects from enhanced light-matter interaction | - Strain limited to ~0.15%  - Requirement of hBN encapsulation for mechanical and electrical stability |  |

**
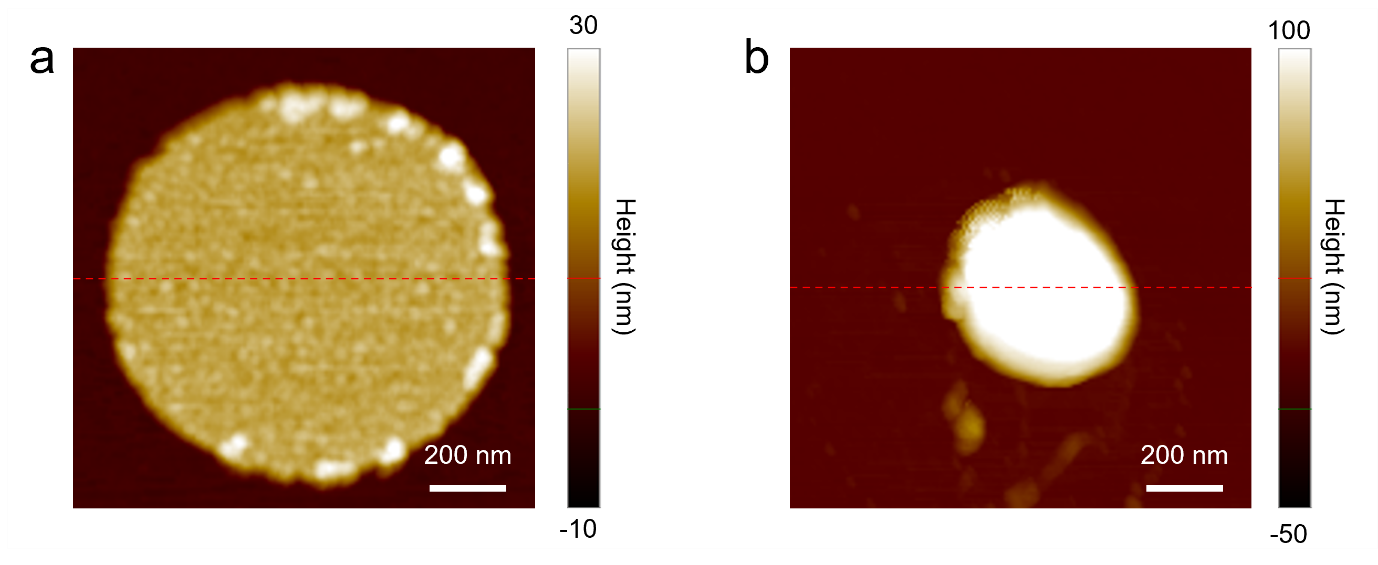
**

**Figure S1.** AFM topography images of (a) as-deposited and (b) recrystallized gold nanopillar.

**
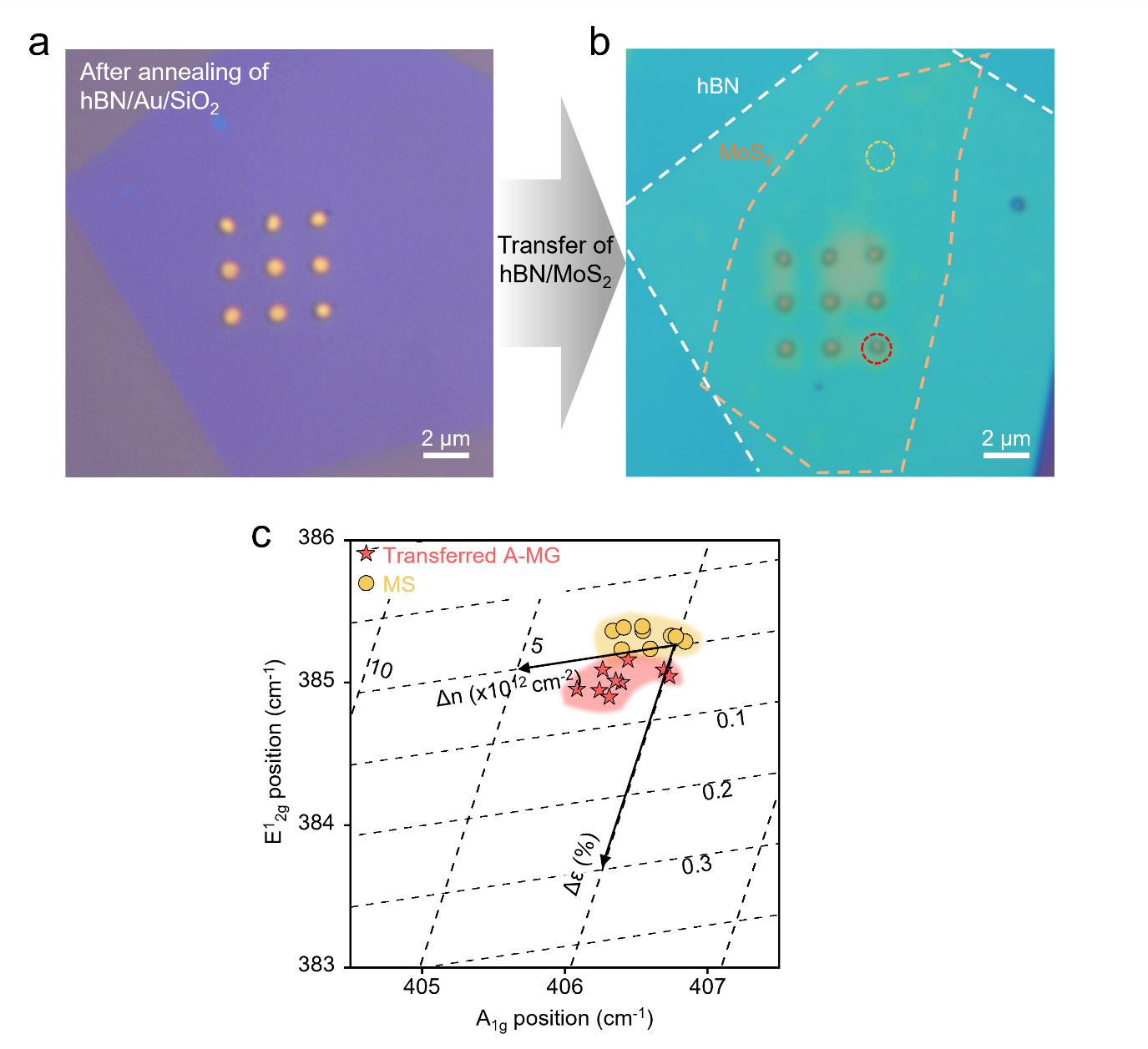
**

**Figure S2.** Optical images of (a) the hBN/Au/SiO_2_ structure after recrystallization and (b) the sample after transferring the top hBN/MoS_2_ onto (a). (c) Plot showing the correlation between E^1^_2g_ and A_1g_ peak positions corresponding to (b).

**
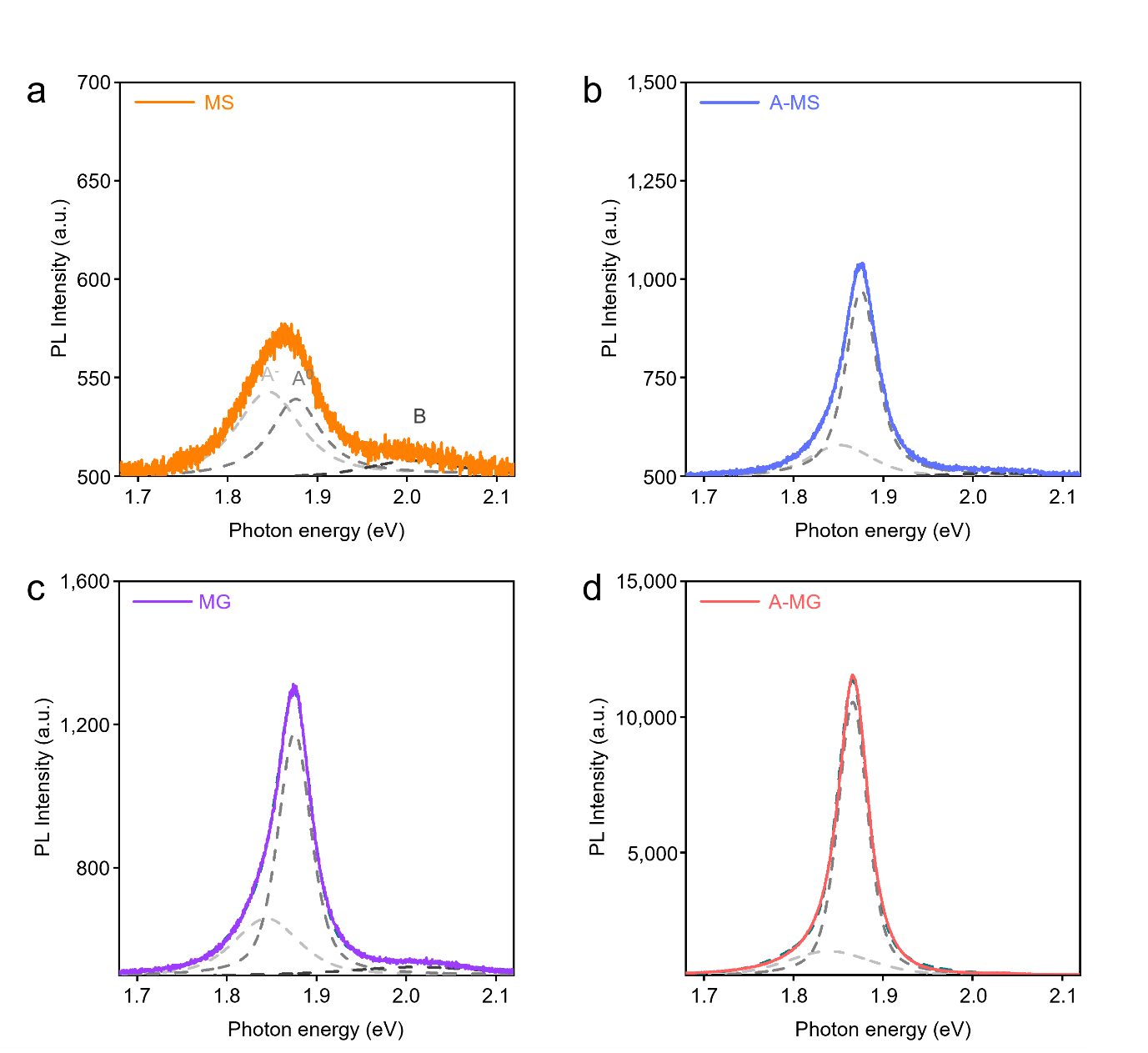
**

**Figure S3.** Deconvolution of the PL spectra for (a) MS, (b) A-MS, (c) MG, and (d) A-MG.

**
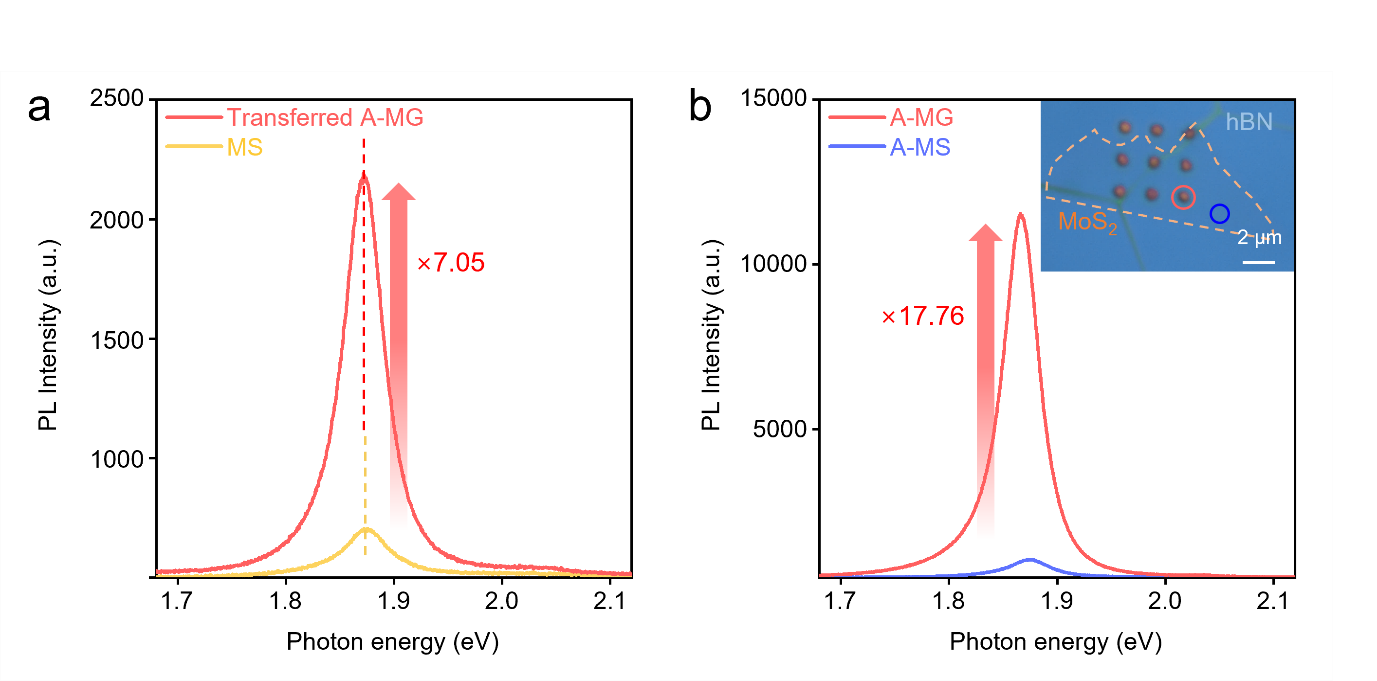
**

**Figure S4.** (a) PL spectra of Transferred A-MG (red) and MS (yellow). (b) PL spectra of A-MG (red) and A-MS (blue).


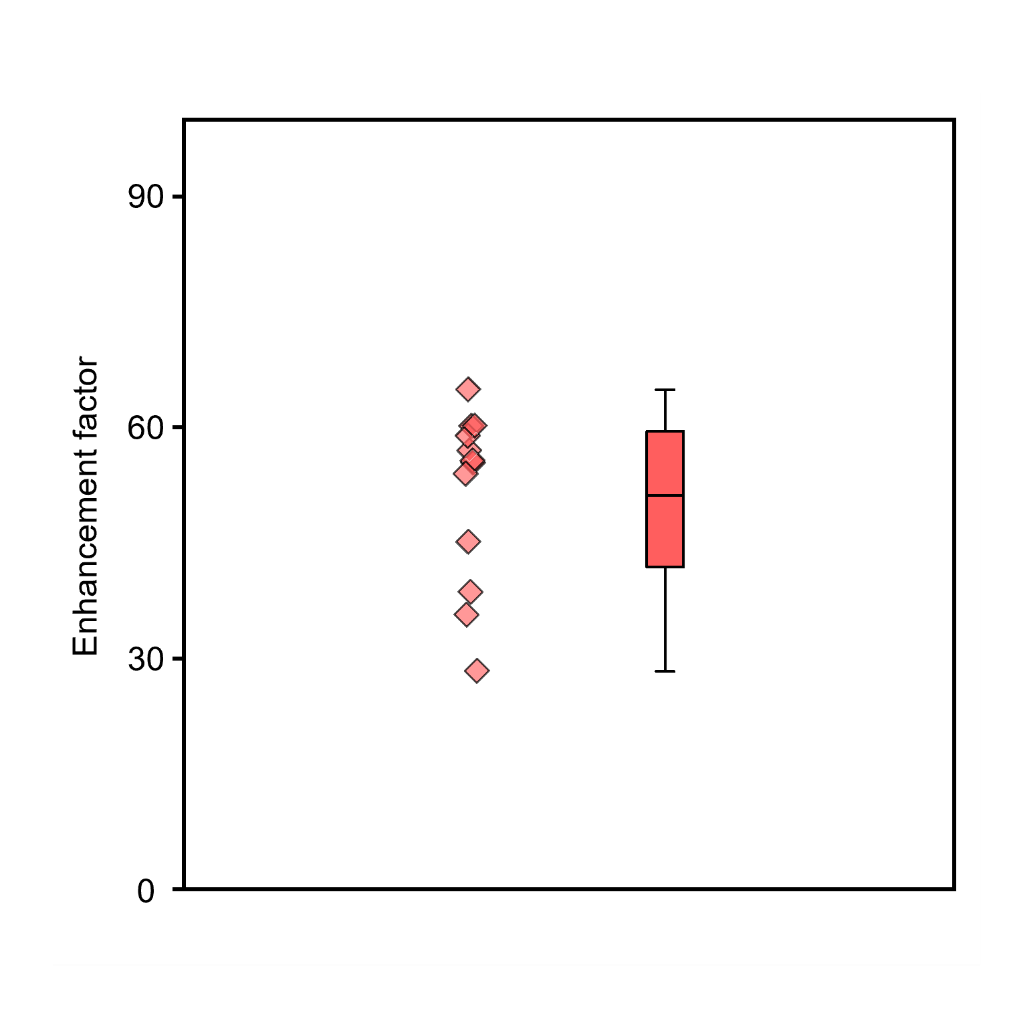


**Figure S5.** PL enhancement factor distribution across multiple batches. Enhancement factors were collected from a total of 12 A-MG samples prepared across two different batches. The center lines in the box plots represent the mean values, while the boxes indicate the interquartile range (25th–75th percentiles) of the dataset. The whiskers denote the minimum and maximum values

**
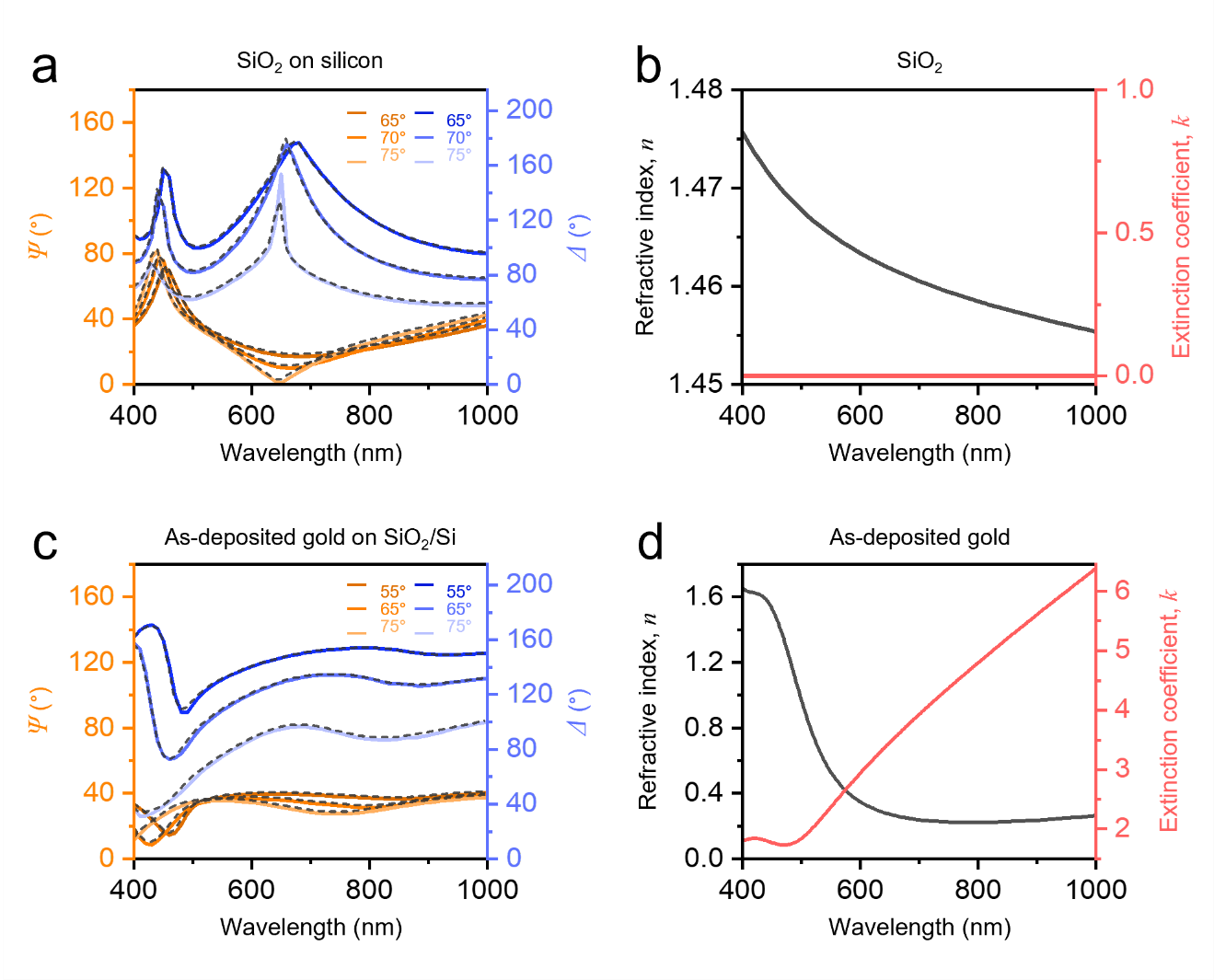
**

**Figure S6.** Ellipsometry data and fitted optical constants used for FDTD simulation. (a) Ellipsometry constants measured from the SiO_2_/Si substrate. (b) Refractive indices and extinction coefficients of the SiO_2_ film. (c) Ellipsometry constants measured from the as-deposited gold film on the SiO_2_/Si substrate. (b) Refractive indices and extinction coefficients of the as-deposited gold film.

**
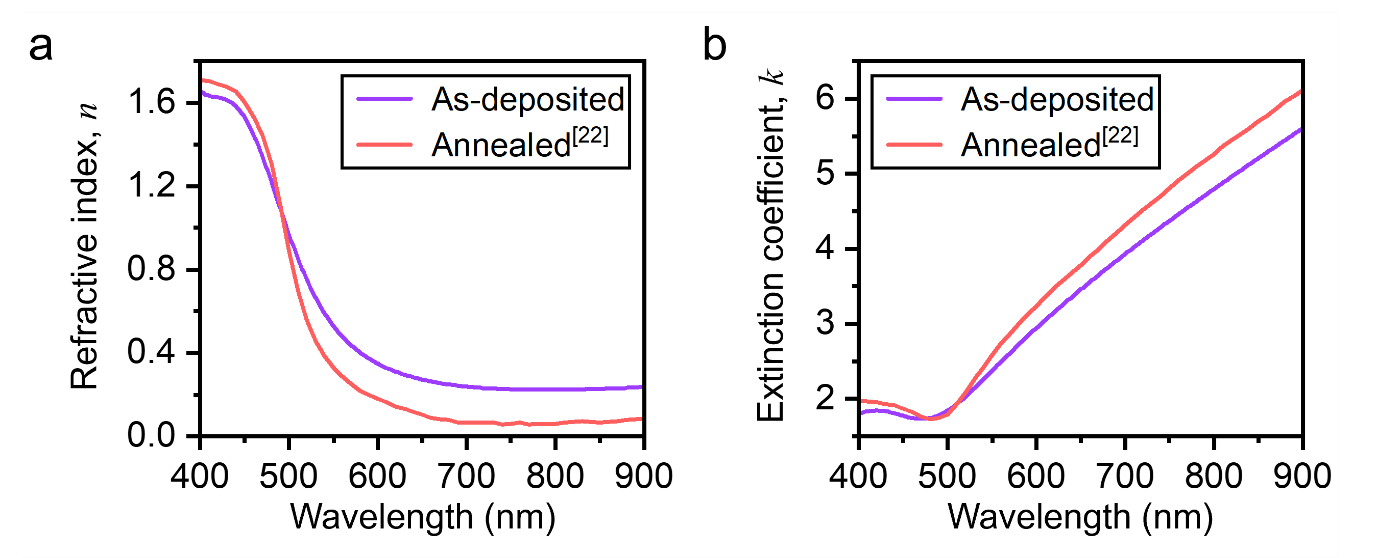
**

**Figure S7.** Comparison of (a) refractive index and (b) extinction coefficient of as-deposited and annealed gold films. For the annealed gold film, the optical constants of single-crystal gold obtained from a previous study^[22]^ are presented because ellipsometry data could not be directly measured from the annealed gold nanopillars due to their size being much smaller than the ellipsometer beam diameter.

**
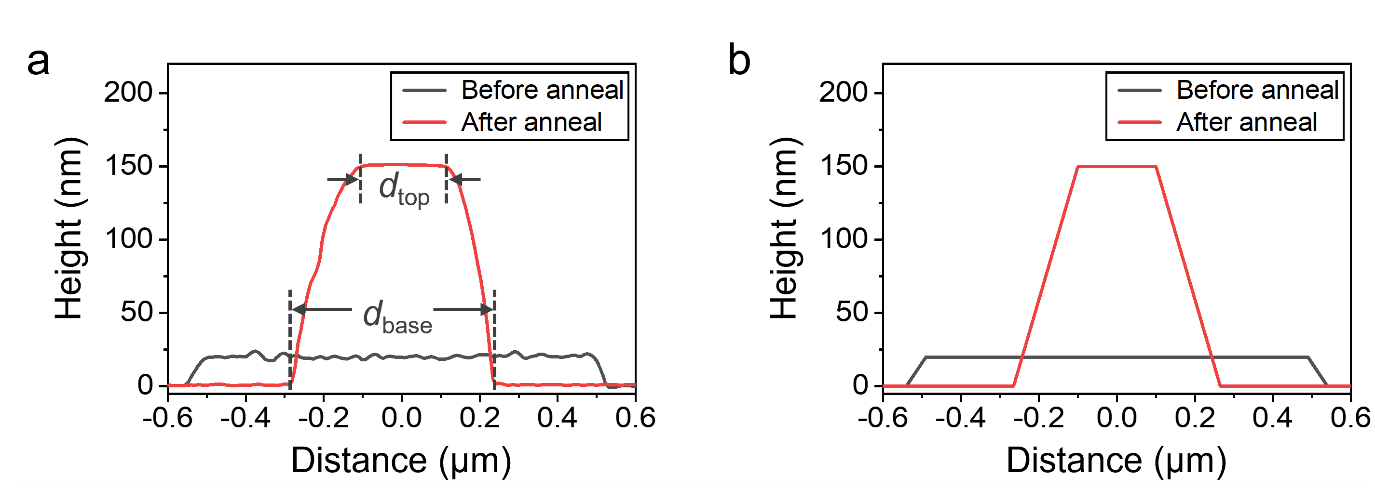
**

**Figure S8.** Structure profile of gold used for FDTD simulations. (a) Height profiles of the gold structure measured by AFM before and after annealing. (b) Height profiles of the gold structure used in FDTD simulation. The based diameter (*d*_base_), top diameter (*d*_top_) and height were extracted from the AFM profiles. For FDTD, the gold structure was approximated as a symmetric cone with a perfectly flat surface and no surface roughness.

**
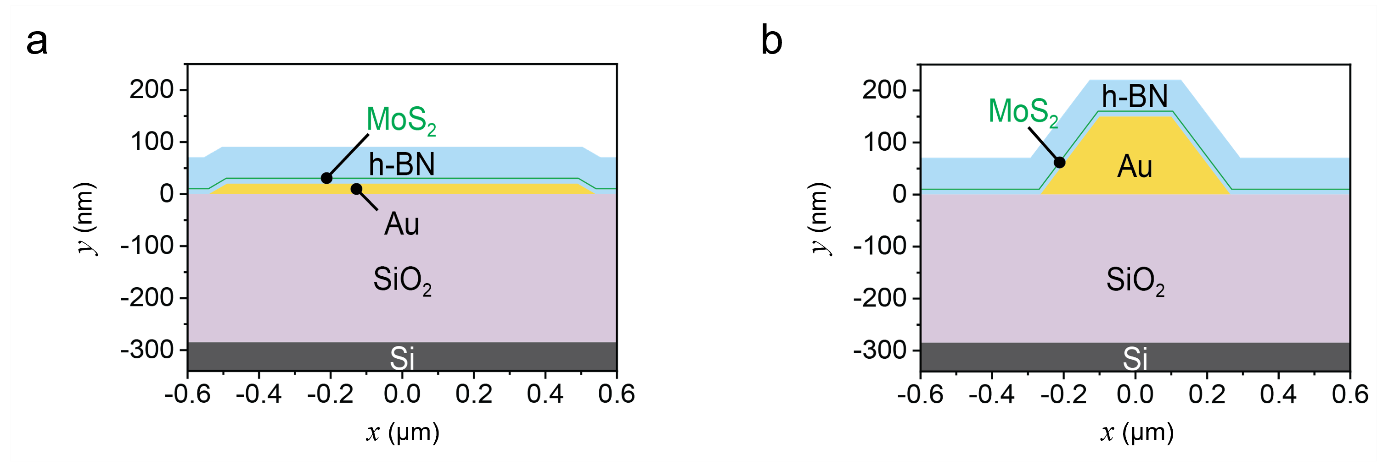
**

**Figure S9.** Overall structure profile of hBN-encapsulated MoS_2_ on gold. (a) Structure profile before annealing and (b) after annealing. The top and bottom hBN layers are approximately 60 nm and 10 nm thick, respectively, and the SiO_2_ substrate is 285 nm thick. The geometry of the gold structure was determined based on AFM measurements. Each layer was assumed to conform perfectly to the underlying surface without any gaps.

**
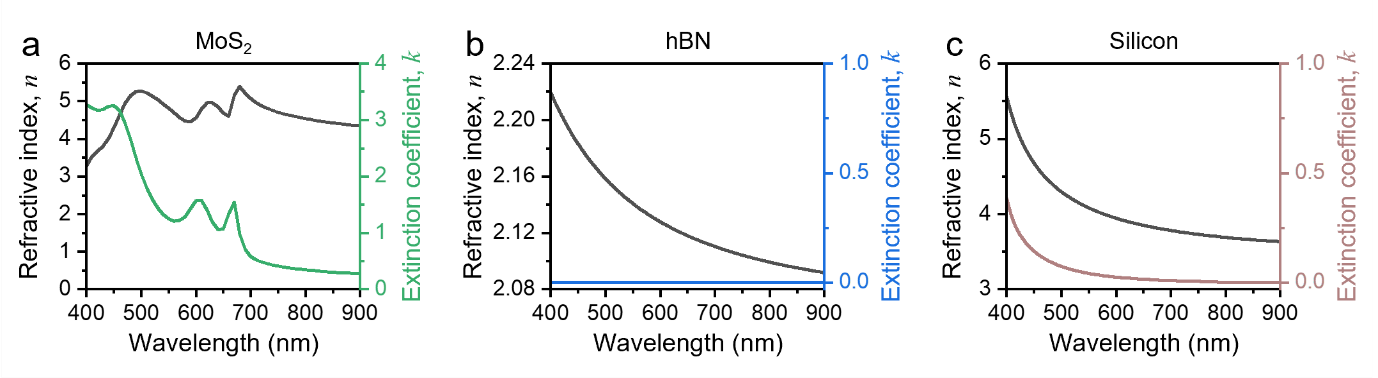
**

**Figure S10.** Optical constants from literature used in the FDTD simulation in this work. (a) MoS_2_, (b) hBN, and (c) silicon.

**Table S1.** Measured and fitted ellipsometry constants of the as-deposited gold film.

| Wavelength (nm) | Measured Ψ | | | Measured Δ | | | Fitted Ψ | | | Fitted Δ | | |
| --- | --- | --- | --- | --- | --- | --- | --- | --- | --- | --- | --- | --- |
|  | 55° | 65° | 75° | 55° | 65° | 75° | 55° | 65° | 75° | 55° | 65° | 75° |
| 400 | 33.2 | 18.0 | 11.2 | 162.0 | 157.6 | 37.0 | 32.5 | 17.6 | 11.2 | 161.1 | 156.3 | 36.1 |
| 410 | 30.9 | 13.0 | 15.2 | 166.6 | 153.6 | 31.5 | 30.4 | 13.0 | 15.4 | 165.4 | 151.1 | 32.1 |
| 420 | 27.8 | 9.4 | 18.7 | 169.8 | 136.9 | 31.4 | 27.5 | 9.8 | 18.6 | 168.5 | 135.3 | 32.5 |
| 430 | 24.1 | 8.7 | 21.7 | 170.8 | 107.4 | 33.2 | 24.1 | 8.9 | 21.3 | 169.7 | 110.1 | 33.6 |
| 440 | 20.2 | 11.0 | 24.3 | 168.1 | 85.1 | 35.6 | 20.3 | 10.6 | 23.9 | 167.9 | 86.9 | 35.0 |
| 450 | 16.5 | 14.7 | 26.6 | 159.1 | 75.5 | 38.4 | 16.4 | 14.2 | 26.5 | 160.0 | 74.3 | 37.3 |
| 460 | 14.5 | 18.7 | 28.6 | 141.5 | 72.9 | 41.5 | 14.0 | 18.8 | 28.9 | 140.5 | 71.0 | 40.7 |
| 470 | 15.9 | 22.8 | 30.5 | 119.8 | 73.5 | 44.8 | 16.1 | 23.5 | 31.0 | 116.9 | 73.3 | 45.0 |
| 480 | 20.9 | 26.8 | 32.3 | 107.3 | 76.8 | 48.6 | 21.9 | 27.3 | 32.5 | 107.9 | 78.6 | 49.7 |
| 490 | 27.1 | 30.1 | 33.7 | 106.8 | 82.2 | 52.9 | 27.3 | 30.1 | 33.6 | 109.5 | 84.5 | 54.2 |
| 500 | 31.6 | 32.4 | 34.6 | 112.2 | 88.2 | 57.3 | 31.0 | 31.9 | 34.2 | 114.1 | 89.9 | 58.3 |
| 510 | 34.1 | 33.8 | 35.2 | 118.0 | 93.6 | 61.4 | 33.3 | 33.1 | 34.7 | 118.6 | 94.5 | 62.0 |
| 520 | 35.5 | 34.6 | 35.5 | 122.3 | 98.1 | 65.1 | 34.8 | 34.0 | 35.0 | 122.4 | 98.5 | 65.4 |
| 530 | 36.5 | 35.2 | 35.6 | 125.8 | 101.9 | 68.5 | 35.8 | 34.7 | 35.2 | 125.5 | 102.0 | 68.5 |
| 540 | 37.2 | 35.7 | 35.7 | 128.6 | 105.2 | 71.6 | 36.6 | 35.3 | 35.4 | 128.1 | 105.1 | 71.5 |
| 550 | 37.8 | 36.1 | 35.7 | 131.0 | 108.3 | 74.5 | 37.3 | 35.7 | 35.4 | 130.4 | 108.0 | 74.4 |
| 560 | 38.3 | 36.4 | 35.5 | 133.2 | 111.0 | 77.3 | 37.8 | 36.0 | 35.4 | 132.6 | 110.7 | 77.1 |
| 570 | 38.7 | 36.6 | 35.4 | 135.2 | 113.6 | 79.9 | 38.3 | 36.3 | 35.3 | 134.5 | 113.2 | 79.7 |
| 580 | 39.0 | 36.7 | 35.1 | 137.1 | 116.0 | 82.4 | 38.6 | 36.5 | 35.1 | 136.3 | 115.5 | 82.2 |
| 590 | 39.3 | 36.8 | 34.8 | 138.8 | 118.3 | 84.7 | 38.9 | 36.6 | 34.8 | 138.0 | 117.7 | 84.5 |
| 600 | 39.5 | 36.8 | 34.4 | 140.3 | 120.4 | 86.9 | 39.1 | 36.6 | 34.4 | 139.6 | 119.8 | 86.8 |
| 610 | 39.6 | 36.7 | 34.0 | 141.8 | 122.3 | 88.9 | 39.3 | 36.6 | 34.0 | 141.1 | 121.8 | 88.8 |
| 620 | 39.7 | 36.6 | 33.4 | 143.2 | 124.1 | 90.8 | 39.4 | 36.5 | 33.5 | 142.5 | 123.6 | 90.7 |
| 630 | 39.7 | 36.4 | 32.8 | 144.4 | 125.8 | 92.4 | 39.4 | 36.3 | 33.0 | 143.8 | 125.3 | 92.4 |
| 640 | 39.7 | 36.2 | 32.2 | 145.6 | 127.3 | 93.8 | 39.4 | 36.1 | 32.4 | 145.0 | 126.9 | 93.8 |
| 650 | 39.7 | 35.9 | 31.6 | 146.7 | 128.7 | 94.9 | 39.4 | 35.8 | 31.7 | 146.1 | 128.3 | 95.0 |
| 660 | 39.6 | 35.7 | 30.9 | 147.7 | 130.0 | 95.7 | 39.3 | 35.6 | 31.0 | 147.2 | 129.6 | 95.9 |
| 670 | 39.5 | 35.3 | 30.2 | 148.7 | 131.1 | 96.3 | 39.2 | 35.2 | 30.4 | 148.1 | 130.7 | 96.5 |
| 680 | 39.4 | 34.9 | 29.6 | 149.5 | 132.0 | 96.5 | 39.1 | 34.9 | 29.7 | 149.0 | 131.7 | 96.7 |
| 690 | 39.2 | 34.6 | 29.0 | 150.3 | 132.8 | 96.4 | 39.0 | 34.5 | 29.1 | 149.8 | 132.5 | 96.7 |
| 700 | 39.1 | 34.2 | 28.5 | 151.0 | 133.4 | 96.0 | 38.8 | 34.1 | 28.6 | 150.5 | 133.1 | 96.4 |
| 710 | 38.9 | 33.8 | 28.0 | 151.6 | 133.8 | 95.4 | 38.7 | 33.7 | 28.2 | 151.1 | 133.6 | 95.8 |
| 720 | 38.7 | 33.4 | 27.7 | 152.2 | 134.1 | 94.6 | 38.5 | 33.4 | 27.8 | 151.7 | 133.9 | 95.1 |
| 730 | 38.5 | 33.1 | 27.5 | 152.6 | 134.2 | 93.7 | 38.3 | 33.0 | 27.6 | 152.1 | 134.0 | 94.2 |
| 740 | 38.3 | 32.7 | 27.4 | 153.0 | 134.3 | 92.7 | 38.1 | 32.7 | 27.5 | 152.5 | 134.1 | 93.2 |
| 750 | 38.2 | 32.4 | 27.4 | 153.3 | 134.2 | 91.6 | 37.9 | 32.4 | 27.5 | 152.8 | 134.0 | 92.2 |
| 760 | 38.0 | 32.1 | 27.5 | 153.5 | 133.9 | 90.6 | 37.8 | 32.1 | 27.6 | 153.0 | 133.8 | 91.1 |
| 770 | 37.8 | 31.9 | 27.7 | 153.7 | 133.6 | 89.5 | 37.6 | 31.8 | 27.8 | 153.2 | 133.4 | 90.1 |
| 780 | 37.7 | 31.7 | 28.1 | 153.9 | 133.2 | 88.7 | 37.4 | 31.6 | 28.1 | 153.4 | 132.9 | 89.2 |
| 790 | 37.5 | 31.5 | 28.5 | 154.0 | 132.5 | 88.0 | 37.2 | 31.4 | 28.6 | 153.5 | 132.1 | 88.4 |
| 800 | 37.3 | 31.5 | 29.0 | 154.0 | 131.6 | 87.4 | 37.0 | 31.4 | 29.1 | 153.5 | 131.2 | 87.9 |
| 810 | 37.1 | 31.6 | 29.5 | 153.9 | 130.7 | 87.0 | 36.8 | 31.4 | 29.6 | 153.4 | 130.2 | 87.5 |
| 820 | 36.8 | 31.6 | 30.1 | 153.7 | 129.5 | 86.7 | 36.6 | 31.6 | 30.2 | 153.2 | 129.3 | 87.4 |
| 830 | 36.7 | 31.9 | 30.7 | 153.2 | 128.4 | 86.7 | 36.4 | 31.9 | 30.8 | 152.8 | 128.4 | 87.4 |
| 840 | 36.6 | 32.3 | 31.3 | 152.8 | 127.9 | 87.1 | 36.3 | 32.3 | 31.4 | 152.3 | 127.7 | 87.7 |
| 850 | 36.6 | 32.8 | 31.9 | 152.2 | 127.7 | 87.5 | 36.2 | 32.7 | 32.0 | 151.6 | 127.1 | 88.0 |
| 860 | 36.6 | 33.3 | 32.4 | 151.6 | 127.4 | 88.1 | 36.3 | 33.2 | 32.6 | 151.0 | 126.7 | 88.5 |
| 870 | 36.7 | 33.7 | 33.0 | 151.0 | 127.1 | 88.6 | 36.4 | 33.7 | 33.1 | 150.3 | 126.4 | 89.1 |
| 880 | 36.9 | 34.2 | 33.5 | 150.4 | 126.5 | 88.8 | 36.6 | 34.2 | 33.6 | 149.8 | 126.3 | 89.7 |
| 890 | 37.1 | 34.7 | 34.0 | 149.9 | 126.5 | 89.5 | 36.8 | 34.7 | 34.1 | 149.3 | 126.4 | 90.4 |
| 900 | 37.4 | 35.2 | 34.4 | 149.5 | 126.8 | 90.2 | 37.1 | 35.2 | 34.6 | 148.9 | 126.5 | 91.2 |
| 910 | 37.8 | 35.7 | 34.9 | 149.3 | 127.2 | 91.2 | 37.4 | 35.7 | 35.0 | 148.6 | 126.8 | 92.0 |
| 920 | 38.1 | 36.2 | 35.3 | 149.1 | 127.5 | 92.1 | 37.8 | 36.1 | 35.4 | 148.4 | 127.1 | 92.8 |
| 930 | 38.4 | 36.7 | 35.7 | 149.1 | 128.2 | 93.7 | 38.1 | 36.5 | 35.8 | 148.3 | 127.5 | 93.6 |
| 940 | 38.7 | 37.1 | 36.0 | 149.1 | 128.8 | 94.6 | 38.4 | 36.9 | 36.1 | 148.3 | 127.9 | 94.4 |
| 950 | 39.1 | 37.5 | 36.4 | 149.2 | 129.3 | 95.7 | 38.7 | 37.3 | 36.4 | 148.4 | 128.4 | 95.3 |
| 960 | 39.4 | 37.8 | 36.7 | 149.2 | 129.8 | 96.7 | 39.1 | 37.7 | 36.7 | 148.5 | 128.9 | 96.1 |
| 970 | 39.7 | 38.1 | 37.0 | 149.4 | 130.3 | 97.5 | 39.3 | 38.0 | 37.0 | 148.7 | 129.4 | 97.0 |
| 980 | 39.9 | 38.5 | 37.2 | 149.6 | 130.8 | 98.3 | 39.6 | 38.3 | 37.3 | 148.9 | 130.0 | 97.8 |
| 990 | 40.2 | 38.7 | 37.5 | 149.9 | 131.4 | 99.2 | 39.9 | 38.6 | 37.5 | 149.2 | 130.5 | 98.7 |
| 1000 | 40.4 | 39.0 | 37.7 | 150.0 | 131.8 | 100.1 | 40.1 | 38.8 | 37.7 | 149.4 | 131.1 | 99.5 |

**Table S2.** Refractive indices and extinction coefficients of the as-deposited gold film.

| Wavelength (nm) | *n* | *k* |  | Wavelength (nm) | *n* | *k* |  | Wavelength (nm) | *n* | *k* |
| --- | --- | --- | --- | --- | --- | --- | --- | --- | --- | --- |
| 400 | 1.657 | 1.804 |  | 605 | 0.338 | 2.998 |  | 810 | 0.223 | 4.881 |
| 405 | 1.642 | 1.821 |  | 610 | 0.328 | 3.050 |  | 815 | 0.224 | 4.922 |
| 410 | 1.634 | 1.835 |  | 615 | 0.319 | 3.103 |  | 820 | 0.224 | 4.963 |
| 415 | 1.629 | 1.843 |  | 620 | 0.311 | 3.156 |  | 825 | 0.225 | 5.004 |
| 420 | 1.625 | 1.847 |  | 625 | 0.303 | 3.207 |  | 830 | 0.225 | 5.045 |
| 425 | 1.621 | 1.844 |  | 630 | 0.296 | 3.258 |  | 835 | 0.226 | 5.085 |
| 430 | 1.614 | 1.836 |  | 635 | 0.289 | 3.309 |  | 840 | 0.226 | 5.126 |
| 435 | 1.602 | 1.823 |  | 640 | 0.283 | 3.359 |  | 845 | 0.227 | 5.167 |
| 440 | 1.584 | 1.808 |  | 645 | 0.278 | 3.409 |  | 850 | 0.228 | 5.207 |
| 445 | 1.559 | 1.790 |  | 650 | 0.272 | 3.458 |  | 855 | 0.228 | 5.247 |
| 450 | 1.528 | 1.773 |  | 655 | 0.268 | 3.507 |  | 860 | 0.229 | 5.287 |
| 455 | 1.490 | 1.758 |  | 660 | 0.264 | 3.556 |  | 865 | 0.230 | 5.328 |
| 460 | 1.446 | 1.746 |  | 665 | 0.260 | 3.604 |  | 870 | 0.231 | 5.368 |
| 465 | 1.396 | 1.737 |  | 670 | 0.256 | 3.651 |  | 875 | 0.232 | 5.408 |
| 470 | 1.341 | 1.734 |  | 675 | 0.252 | 3.699 |  | 880 | 0.233 | 5.448 |
| 475 | 1.282 | 1.736 |  | 680 | 0.249 | 3.746 |  | 885 | 0.234 | 5.488 |
| 480 | 1.221 | 1.745 |  | 685 | 0.246 | 3.792 |  | 890 | 0.235 | 5.527 |
| 485 | 1.158 | 1.761 |  | 690 | 0.244 | 3.839 |  | 895 | 0.236 | 5.567 |
| 490 | 1.094 | 1.783 |  | 695 | 0.241 | 3.885 |  | 900 | 0.237 | 5.607 |
| 495 | 1.031 | 1.812 |  | 700 | 0.239 | 3.930 |  | 905 | 0.238 | 5.646 |
| 500 | 0.970 | 1.845 |  | 705 | 0.237 | 3.976 |  | 910 | 0.239 | 5.685 |
| 505 | 0.910 | 1.885 |  | 710 | 0.235 | 4.021 |  | 915 | 0.240 | 5.724 |
| 510 | 0.854 | 1.930 |  | 715 | 0.233 | 4.066 |  | 920 | 0.242 | 5.764 |
| 515 | 0.801 | 1.978 |  | 720 | 0.232 | 4.111 |  | 925 | 0.243 | 5.803 |
| 520 | 0.752 | 2.030 |  | 725 | 0.230 | 4.156 |  | 930 | 0.244 | 5.842 |
| 525 | 0.706 | 2.084 |  | 730 | 0.229 | 4.199 |  | 935 | 0.246 | 5.881 |
| 530 | 0.664 | 2.141 |  | 735 | 0.228 | 4.243 |  | 940 | 0.247 | 5.920 |
| 535 | 0.626 | 2.198 |  | 740 | 0.227 | 4.287 |  | 945 | 0.248 | 5.959 |
| 540 | 0.591 | 2.256 |  | 745 | 0.226 | 4.330 |  | 950 | 0.249 | 5.998 |
| 545 | 0.559 | 2.315 |  | 750 | 0.225 | 4.374 |  | 955 | 0.251 | 6.036 |
| 550 | 0.530 | 2.373 |  | 755 | 0.225 | 4.417 |  | 960 | 0.253 | 6.075 |
| 555 | 0.503 | 2.433 |  | 760 | 0.224 | 4.460 |  | 965 | 0.254 | 6.114 |
| 560 | 0.480 | 2.491 |  | 765 | 0.224 | 4.503 |  | 970 | 0.255 | 6.152 |
| 565 | 0.457 | 2.550 |  | 770 | 0.223 | 4.546 |  | 975 | 0.257 | 6.191 |
| 570 | 0.437 | 2.608 |  | 775 | 0.223 | 4.588 |  | 980 | 0.258 | 6.230 |
| 575 | 0.419 | 2.665 |  | 780 | 0.223 | 4.630 |  | 985 | 0.260 | 6.268 |
| 580 | 0.402 | 2.722 |  | 785 | 0.223 | 4.672 |  | 990 | 0.262 | 6.306 |
| 585 | 0.387 | 2.778 |  | 790 | 0.223 | 4.714 |  | 995 | 0.263 | 6.345 |
| 590 | 0.373 | 2.834 |  | 795 | 0.223 | 4.756 |  | 1000 | 0.265 | 6.383 |
| 595 | 0.361 | 2.889 |  | 800 | 0.223 | 4.797 |  |  |  |  |
| 600 | 0.349 | 2.944 |  | 805 | 0.223 | 4.839 |  |  |  |  |

**
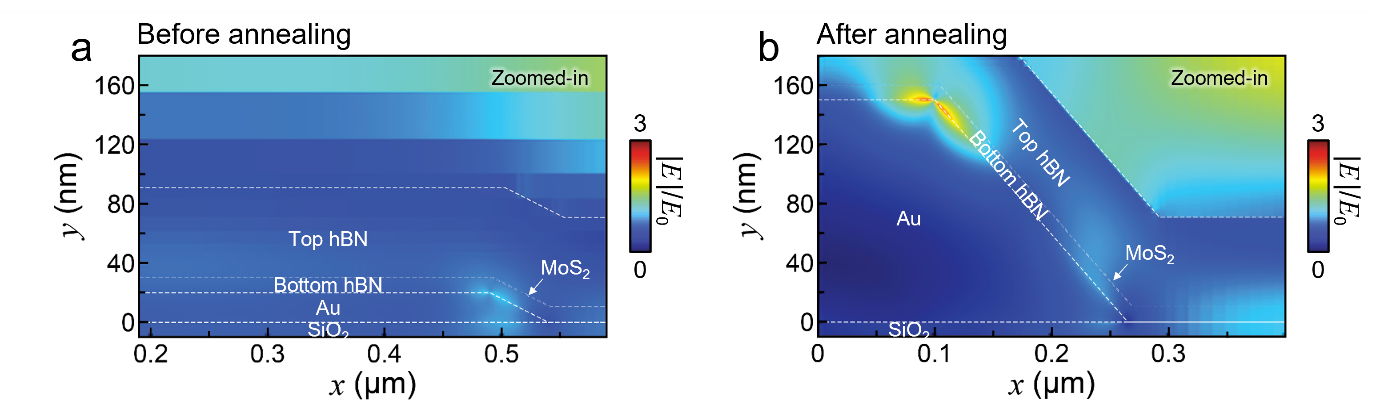
**

**Figure S11.** Zoomed-in spatial maps of the electric field distribution calculated using finite-difference time-domain (FDTD) simulation for the hBN/MoS_2_/hBN/Au/SiO_2_ structure: (a) before and (b) after annealing.


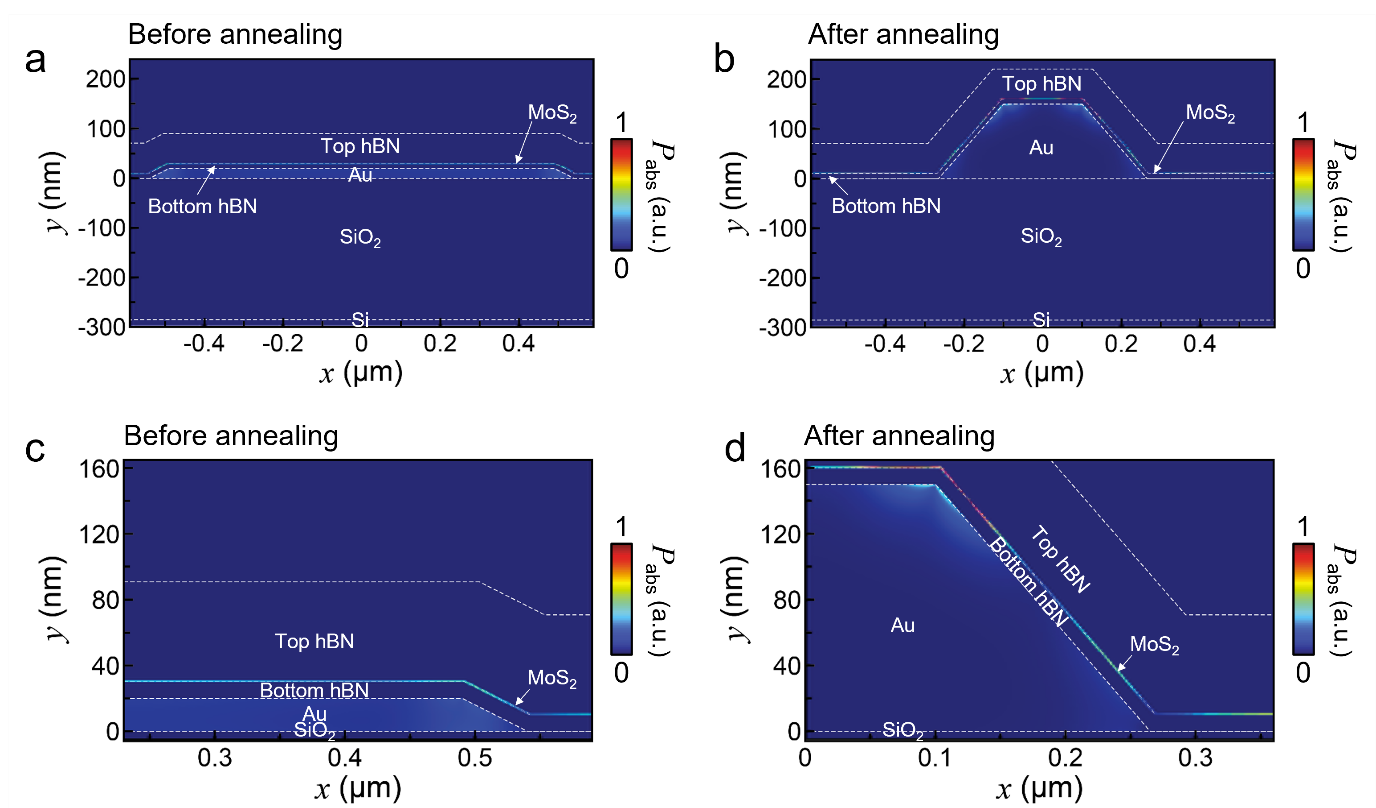


**Figure S12.** Spatial distribution of the light power absorption per unit volume (*P*_abs_) for the hBN/MoS_2_/hBN/Au/SiO_2_ structure: (a) before and (b) after annealing. Zoomed-in images showing the spatial *P*_abs_ distributions (c) before and (d) after annealing.


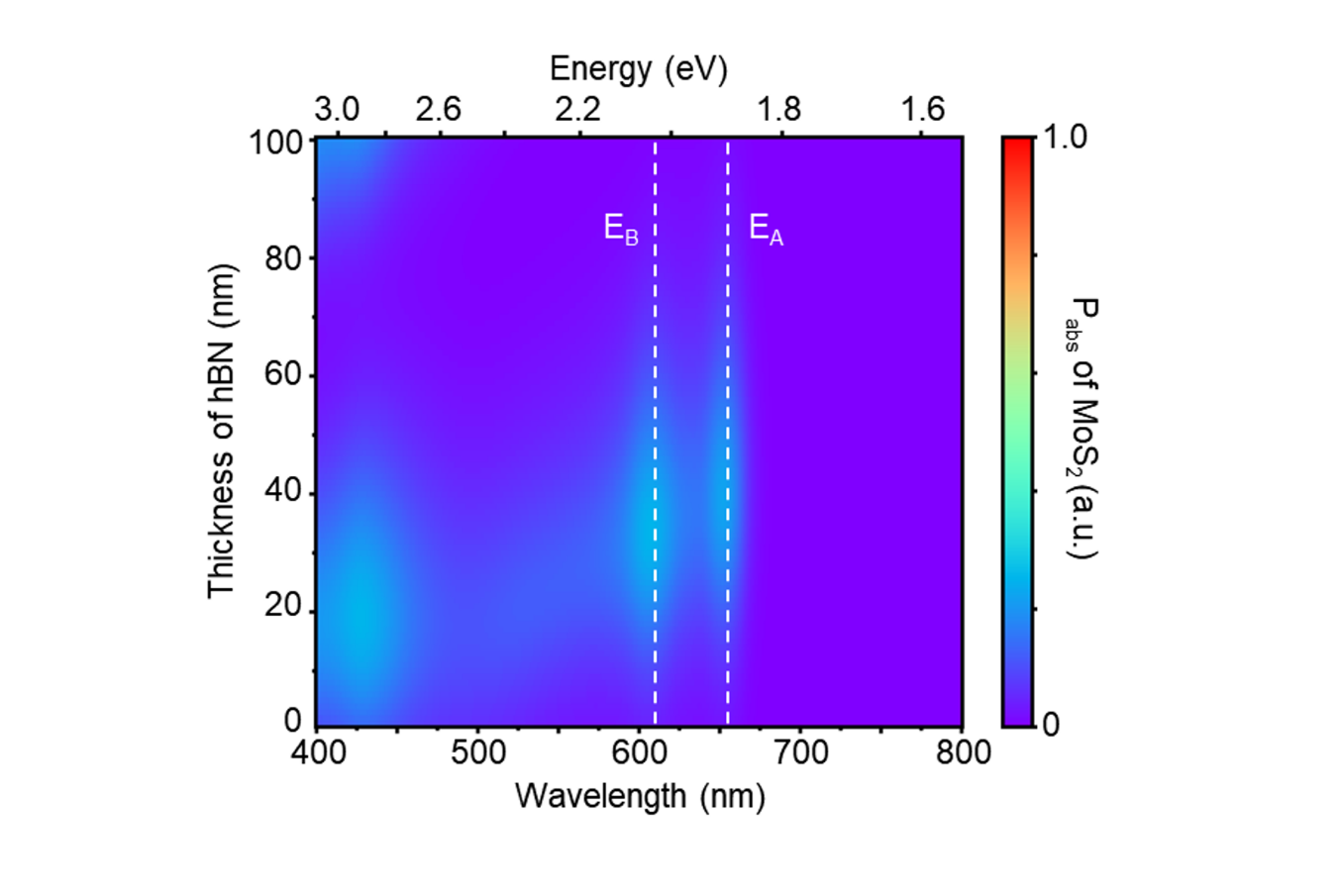


**Figure S13.** Calculated absorbance in the MoS₂ layer as a function of bottom hBN thickness using TMM simulation.


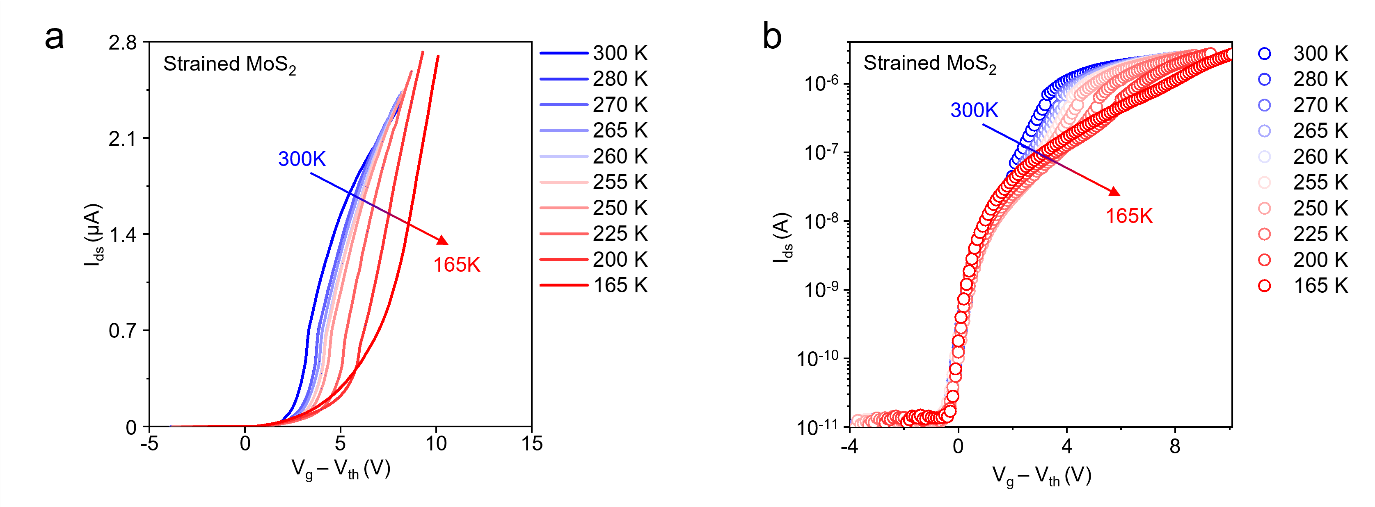


**Figure S14.** Temperature-dependent transfer curves of strained MoS_2_ shown in Figure 6, presented in (a) linear and (b) logarithmic scales.

**
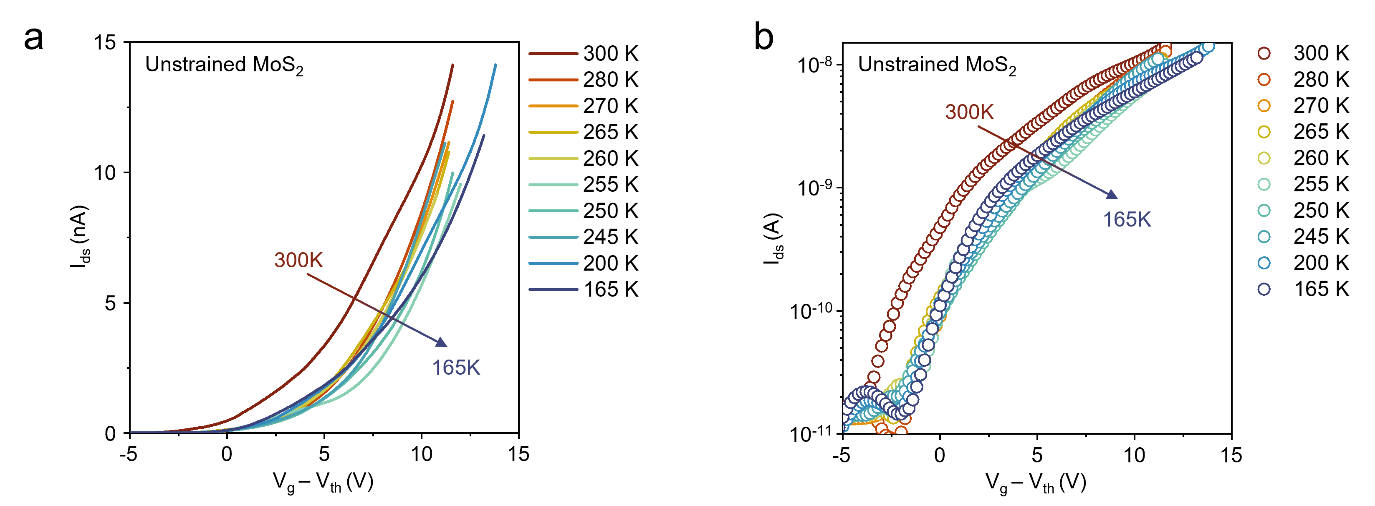
**

**Figure S15.** Temperature-dependent transfer curves of unstrained MoS_2_, presented in (a) linear and (b) logarithmic scales at V_ds_ = 0.5 V.

**
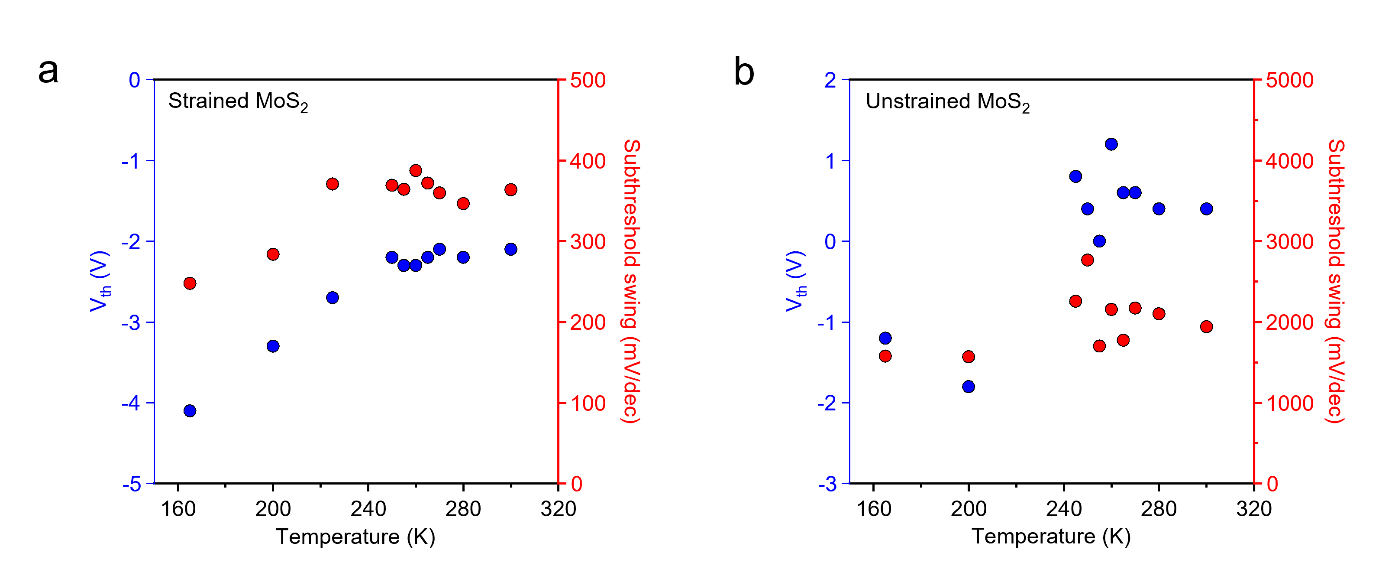
**

**Figure S16.** Temperature dependence of the subthreshold swing (SS) and threshold voltage (V_th_) for the (a) strained and (b) unstrained MoS_2_ shown in Figure S14 and Figure S15.

**Reference**

[1] Y. Cai, J. Lan, G. Zhang, Y.-W. Zhang, *Phys. Rev. B.* **2014**, *89*, 035438.

[2] B. Chakraborty, A. Bera, D. Muthu, S. Bhowmick, U. V. Waghmare, A. Sood, *Phys. Rev. B.* **2012**, *85*, 161403.

[3] H. J. Conley, B. Wang, J. I. Ziegler, R. F. Haglund Jr, S. T. Pantelides, K. I. Bolotin, *Nano Lett.* **2013**, *13*, 3626.

[4] H.-Y. Park, S. R. Dugasani, D.-H. Kang, J. Jeon, S. K. Jang, S. Lee, Y. Roh, S. H. Park, J.-H. Park, *ACS Nano* **2014**, *8*, 11603.

[5] C. Rice, R. Young, R. Zan, U. Bangert, D. Wolverson, T. Georgiou, R. Jalil, K. Novoselov, *Phys. Rev. B.* **2013**, *87*, 081307.

[6] A. Tarasov, S. Zhang, M.-Y. Tsai, P. M. Campbell, S. Graham, S. Barlow, S. R. Marder, E. M. Vogel, *Adv. Mater.* **2015**, *27*, 1175.

[7] J. Wu, S. Zhang, L. Tong, J. Zhang, *Raman Spectroscopy of Two-Dimensional Materials* **2018**, *276*, 53.

[8] D. Lloyd, X. Liu, J. W. Christopher, L. Cantley, A. Wadehra, B. L. Kim, B. B. Goldberg, A. K. Swan, J. S. Bunch, *Nano Lett.* **2016**, *16*, 5836.

[9] A. Michail, N. Delikoukos, J. Parthenios, C. Galiotis, K. Papagelis, *Appl. Phys. Lett.* **2016**, *108*.

[10] Y. Zhang, H. L. Zhao, S. Huang, M. A. Hossain, A. M. van der Zande, *ACS Nano* **2024**, *18*, 12377.

[11] M. Jaikissoon, Ç. Köroğlu, J. A. Yang, K. Neilson, K. C. Saraswat, E. Pop, *Nat. Electron.* **2024**, *7*, 885.

[12] T. Peña, S. A. Chowdhury, A. Azizimanesh, A. Sewaket, H. Askari, S. M. Wu, *2D Materials* **2021**, *8*, 045001.

[13] I. M. Datye, A. Daus, R. W. Grady, K. Brenner, S. Vaziri, E. Pop, *Nano Lett.* **2022**, *22*, 8052.

[14] Z. Li, Y. Lv, L. Ren, J. Li, L. Kong, Y. Zeng, Q. Tao, R. Wu, H. Ma, B. Zhao, *Nat. Commun.* **2020**, *11*, 1151.

[15] H. Shin, A. K. Katiyar, A. T. Hoang, S. M. Yun, B. J. Kim, G. Lee, Y. Kim, J. Lee, H. Kim, J.-H. Ahn, *ACS Nano* **2024**, *18*, 4414.

[16] H. Li, A. W. Contryman, X. Qian, S. M. Ardakani, Y. Gong, X. Wang, J. M. Weisse, C. H. Lee, J. Zhao, P. M. Ajayan, *Nat. Commun.* **2015**, *6*, 7381.

[17] X. Liu, B. Erbas, A. Conde-Rubio, N. Rivano, Z. Wang, J. Jiang, S. Bienz, N. Kumar, T. Sohier, M. Penedo, *Nat. Commun.* **2024**, *15*, 6934.

[18] H. K. Ng, D. Xiang, A. Suwardi, G. Hu, K. Yang, Y. Zhao, T. Liu, Z. Cao, H. Liu, S. Li, *Nat. Electron.* **2022**, *5*, 489.

[19] Y. Y. Hui, X. Liu, W. Jie, N. Y. Chan, J. Hao, Y.-T. Hsu, L.-J. Li, W. Guo, S. P. Lau, *ACS Nano* **2013**, *7*, 7126.

[20] A. Varghese, A. H. Pandey, P. Sharma, Y. Yin, N. V. Medhekar, S. Lodha, *Nano Lett.* **2024**, *24*, 8472.

[21] G. Plechinger, A. Castellanos-Gomez, M. Buscema, H. S. Van Der Zant, G. A. Steele, A. Kuc, T. Heine, C. Schueller, T. Korn, *2D Materials* **2015**, *2*, 015006.

[22] K. Ikeda, S. Suzuki, K. Uosaki, *J. Am. Chem. Soc.* **2013**, *135*, 17387.
